# Elevated IgE promotes cardiac fibrosis by suppressing miR-486a-5p

**DOI:** 10.7150/thno.47845

**Published:** 2021-06-05

**Authors:** Hongmei Zhao, Hongqin Yang, Chi Geng, Yang Chen, Yaqin Tang, Zhiwei Li, Junling Pang, Ting Shu, Yu Nie, Yongshuo Liu, Kegang Jia, Jing Wang

**Affiliations:** 1 State Key Laboratory of Medical Molecular Biology, Institute of Basic Medical Sciences, Chinese Academy of Medical Sciences, Department of Pathophysiology, Peking Union Medical College, Beijing 100005, China.; 2 State Key Laboratory of Cardiovascular Disease, Fuwai Hospital, National Center for Cardiovascular Disease, Chinese Academy of Medical Sciences and Peking Union Medical College, Beijing 102308, China.; 3 Biomedical Pioneering Innovation Center (BIOPIC), Beijing Advanced Innovation Center for Genomics, Peking-Tsinghua Center for Life Sciences, Peking University Genome Editing Research Center, State Key Laboratory of Protein and Plant Gene Research, School of Life Sciences, Peking University, Beijing 100871, China.; 4 Department of Clinical Laboratory, Binzhou Medical University Hospital, Binzhou, Shandong 256603, China.; 5 Department of Clinical Laboratory, TEDA International Cardiovascular Hospital, Tianjin 300457, China.

**Keywords:** cardiac fibrosis, IgE, FcεR1, microRNAs, cardiac fibroblasts

## Abstract

**Rationale:** Cardiac fibrosis is an important feature of cardiac remodeling and is a hallmark of heart failure. Recent studies indicate that elevated IgE plays a causal role in pathological cardiac remodeling. However, the underlying mechanism of how IgE promotes cardiac fibrosis has not been fully elucidated.

**Methods and Results:** To explore the function of IgE in cardiac fibrosis, we stimulated mouse primary cardiac fibroblasts (CFs) with IgE and found that both IgE receptor (FcεR1) and fibrosis related proteins were increased after IgE stimulation. Specific deletion of FcεR1 in CFs alleviated angiotensin II (Ang II)-induced cardiac fibrosis in mice. To investigate the mechanisms underlying the IgE-mediated cardiac fibrosis, deep miRNA-seq was performed. Bioinformatics and signaling pathway analysis revealed that IgE upregulated *Col1a1* and *Col3a1* expression in CFs by repressing miR-486a-5p, with *Smad1* participating downstream of miR-486a-5p in this process. Lentivirus-mediated overexpression of miR-486a-5p was found to alleviate Ang II-induced myocardial interstitial fibrosis in mice. Moreover, miR-486-5p serum levels were lower in patients with heart failure than in healthy controls, and were negatively correlated with NT-proBNP levels.

**Conclusions:** Our study demonstrates that elevated IgE promotes pathological cardiac fibrosis by modulating miR-486a-5p and downstream factors, such as *Smad1*. These findings suggest new targets for pathological cardiac fibrosis intervention.

## Introduction

Cardiac fibrosis is an integral component of most myocardial diseases. The hallmark of cardiac fibrosis is excessive deposition of extracellular matrix (ECM) in the cardiac interstitium, which contributes to increased myocardium passive stiffness and progressively worsening cardiac function that can ultimately lead to lethal arrhythmias and heart failure (HF) 1. Cardiac fibroblasts (CFs) are a major heart cell population and are responsible for ECM homeostasis. In response to pathologic stress and environmental stimuli, they transform into myofibroblasts, thereby altering matrix generation and degradation and contributing to cardiac fibrosis 2, 3. To date, clinically safe and effective therapies for cardiac fibrosis are lacking 4. Thus, novel regulators and potential therapeutic targets need to be identified to facilitate clinical intervention.

The molecular mechanisms underlying myofibroblast activation and fibrosis are complicated and involve many humoral factors including the renin/angiotensin/aldosterone system, inflammatory cytokines and chemokines, endothelin-1, and growth factors such as transforming growth factor β (TGF-β) and platelet-derived growth factor (PDGF) 1, 5. IgE is one of five classes of immunoglobins and they mainly function in allergic reactions 6. It has been previously reported that high IgE levels are associated with several types of cardiovascular disorders, including abdominal aortic aneurysm 7, 8, atherosclerosis 9-11, atherosclerotic cardiovascular disease (ASCVD) 12, 13, and HF 14. Our recent study revealed a crucial role for IgE and its high affinity receptor (FcεR1) in promoting myocardial interstitial fibrosis and ventricular remodeling 14. We found that IgE could directly activate primary rat CFs and promote matrix protein production in an FcεR1-dependent manner, while identifying TGF-β as a critical mediator of this process 14. It has become clear that microRNAs (miRNAs) also play key roles in regulating cardiac fibrosis 15, 16. Evidence shows that several miRNAs (miR-10a, miR-675, and miR-135a) are involved in regulating TGF-β/SMADs signaling, thereby modulating ECM production 17-19. Recent studies have also reported that IgE alters miRNA expression profiles 20-22. Therefore, the role of miRNAs and the specific contribution and molecular mechanism of CFs in IgE-induced cardiac fibrosis deserve further investigation.

In this study, we produced CF-specific FcεR1 knockout (KO) mice to elucidate the contribution of CFs in IgE-FcεR1-induced cardiac fibrosis. To explore the role of miRNAs in IgE-induced cardiac fibrosis, we performed unbiased miRNA deep sequencing analyses and discovered a novel signaling pathway (IgE/FcεR1/miR-486a-5p/*Smad1*) that acts in IgE-mediated CF activation *in vitro* and cardiac fibrosis *in vivo*. We also defined a relationship between serum miR-486-5p levels and human HF. Taken together, these findings point to IgE-FcεR1 and miR-486a-5p as potential, novel therapeutic targets for the treatment of cardiac fibrosis.

## Materials and methods

### Primary mouse cardiac fibroblasts isolation and culture

CFs were isolated from 2- to 4-week-old mice because young mouse CFs can be maintained for several passages, yielding sufficient cells for experiments within 3-4 passages; whereas, CFs isolated from adult mice rapidly senesce, cease replication, or undergo phenotypic modulation 23. Primary CFs were isolated as previously described 24. In brief, mice were anesthetized with nembutal, transferred to a surgical area, and fixed on a dissecting board. After wiping the chest with 70% ethanol, hearts were harvested, placed in a 10 cm petri dish containing phosphate buffered saline (PBS) at room temperature, and cut into small pieces (less than 1 mm^3^) followed by removal of extraneous tissues (auricle and the ascending aorta). The small pieces of heart tissue were enzymatically digested in 3 mL of Hanks's medium with 2.5% trypsin plus 2 μg/mL collagenase II at 37℃ with agitation for 5 min. Then, the supernatant was transferred to equal amounts of DMEM supplemented with 10% FBS to stop the digestion. The trypsin digestion was repeated (two or three times) until the tissues were entirely digested. Cells were collected and passed through a 70 μm strainer to remove tissue debris and obtain single cell suspension. Then, cells were spun down at 150Xg for 5 min. After resuspension, cells were seeded on a fresh 10 cm culture dish and incubated at 37℃ and 5% CO_2_ for 60 min in DMEM containing 10% FBS. Finally, the supernatant was removed and adherent CFs were further cultured in DMEM containing 10% FBS for 72 h. To analyze IgE-induced CF activation, CFs were isolated from FcεR1-WT and FcεR1-KO (*Fcer1a^-/-^*) mice (C57BL/6, N9, The Jackson Laboratory, Bar Harbor, ME) and treated with IgE (5 μg/mL) for different times (0, 3, or 24 h).

### RNA isolation and reverse-transcription polymerase chain reaction (RT-PCR)

Total RNA was extracted from fresh left ventricles or cells using Trizol (Cat#15596018, Invitrogen, Carlsbad, CA, USA) in accordance with the manufacturer's instructions. RNA sample concentration and purity were measured with a Nanodrop. First strand cDNA was synthesized by M-MLV reverse transcriptase (Cat# M1705, Promega, USA) using 1-2 μg of total RNA. The mRNA levels of *Fcer1a*, *Col1a1*, *Col3a1*, *Smad1*, *Postn*, *a-SMA*, and miR-486a-5p were analyzed by qPCR. Gene expression was normalized to* Gapdh*, and miRNA expression was normalized to *U6*. Oligonucleotide primers used for mRNA detection are listed in Table S1.

### Western blot

Total protein was extracted from cultured cells or myocardial tissues in RIPA lysis buffer (50 mM Tris, pH 7.4; 150 mM NaCl; 1% NP-40; 0.1% SDS; and 0.1% EDTA) with EDTA-free protease inhibitor cocktail (Cat#04693132001, Roche, NJ, USA) on ice for 30 min and centrifuged at 13,000 rpm for 10 min. Total protein concentrations were determined with a BCA Protein Assay Kit (Cat#23250, Thermo Scientific, Waltham, MA, USA). Equal amounts of proteins (30-50 μg) were separated by 8%-12% standard sodium dodecyl sulfate-polyacrylamide gel electrophoresis and then transferred to a polyvinylidene difluoride membrane (Cat#IPVH00010, Millipore, Billerica, MA, USA). The membrane was blocked in 5% milk/Tris buffered saline-Tween (TBST) for 1 h at room temperature and then incubated with the appropriate primary antibodies at 4 °C overnight. Antibodies, sources, and dilutions were as follows: rabbit anti-FcεR1 antibody (Cat#10980-1-AP, Proteintech, 1:1000), rabbit anti-α-SMA (Cat#ab5694, Abcam, 1:1000), rabbit anti-SMAD1 (Cat#6944S, Cell Signaling, 1:1000), rabbit anti-phospho-SMAD1 (Cat#PA5-36771, Invitrogen, 1:1000), rabbit anti-SMAD2 (Cat#12570-1-AP, Proteintech, 1:1000), rabbit anti-phospho-SMAD2 (Cat#18338S, Cell Signaling, 1:1000), rabbit anti-TGF-β1 (Cat#ab179695, Abcam, 1:1000), rabbit anti-Collagen Ⅰ (Cat#PA1-26204, Invitrogen, 1:1000), rabbit anti-Collagen Ⅲ (Cat#22734-1-AP, Proteintech, 1:1000), rabbit anti-GFP (Cat#ab290, Abcam, 1:1000), and rabbit anti-GAPDH (Cat#10494-1-AP, Proteintech, 1:3000). After three washes with TBST, the membrane was incubated with the corresponding horseradish peroxidase (HRP)-labeled anti-rabbit IgG (Cat#31460, Invitrogen, 1:4000) for 1 h at room temperature. Immunoreactive bands were visualized with Super Signal West Pico Chemiluminescent Substrate (Cat#34580, Thermo Fisher, USA).

### MiRNA deep sequencing

Primary CFs were treated with IgE for 24 h. Small RNA was isolated from total RNA with a miRVana miRNA isolation kit (Cat#AM1561, Invitrogen, USA) in accordance with manufacturer's instructions. Small RNA libraries were generated and miRNA sequencing was performed by Genergy Bio (Genergy Bio-technology Co., Ltd., Shanghai, China) using an Illumina HiSeq 2000 (Illumina, USA). FASTX-Toolkit (http://hannonlab.cshl.edu/fastx_toolkit/index.html) was used to trim the adaptors from the 3-prime end as well as the low-quality reads. The trimmed reads were then mapped to known miRNA sequences in miRBase (release 21) 25 using bowtie software 26. The expression level of each miRNA was calculated as TPM (transcript per million) on the basis of reads counts for each miRNA and the total reads counts for the whole sample. Significant differentially expressed miRNAs were selected on the basis of *t*-tests. Depictions of differentially expressed miRNAs were constructed with R package pheatmap v1.0.12 (https://CRAN.R-project.org/package=pheatmap). Next, we identified miRNAs responsible for fibrosis from the intersection of our miR-Seq results and miRNAs predicted to directly target major fibrotic genes* Col1a1* and *Col3a1* by the Targetscan 7.1 database. Additionally, the candidates were selected by taking the intersection of our miR-seq results and fibrosis-related miRNAs identified with miRNA arrays (Mouse Fibrosis miRNA PCR Array: MIMM-117Z, QIAGEN, https://geneglobe.qiagen.com/product-groups/miscript-mirna-pcr-arrays) and by literature reports 15, 27.

### Dual-luciferase reporter assay

To make pMIR-reporter constructs, the predicted miR-486a-5p binding site within the *Smad1* 3′-UTR was inserted downstream of the firefly luciferase gene. Mutated miR-486a-5p binding sites in the *Smad1* 3′-UTR were created in pMIR-reporter constructs. For miRNA target analysis, 293T cells were co-transfected with 400 ng pMIR-reporter luciferase construct, 10 ng pRL-TK vector, and 50 nM miR-485a-5p mimic or scrambled controls in 24-well plates. After a 24 h transfection, cells were harvested and assayed with a Dual Luciferase Assay (Cat# E1910, Promega, USA) in accordance with the manufacturer's instructions. Firefly luciferase activity for each transfected well was normalized to Renilla luciferase activity. All transfection assays were performed in triplicates.

### Transient transfection

The miR-486a-5p mimics, inhibitors, miRNA-scrambled control, siRNA-scrambled control, and *Smad1* siRNA were obtained from RIBOBIO (Guangzhou, China). The *Smad1* (NM_008539) mouse ORF clone was obtained from Origene (Cat#MR226823, Origene). The day before transfection, CFs were seeded into 24-well plates (1 × 10^5^ cells per well). Transfection was carried out using Lipofectamine 3000 (Cat#L3000015, Invitrogen, Carlsbad, CA, USA) in accordance with the manufacturer's procedure. The expression levels of different RNAs were assayed by qPCR with a Roche 480 sequence Detection System (Roche, NJ, USA). For western blots, CFs (approximately 70% confluent) were seeded into 6-well plates.

### Lentiviral vector construction and cell transduction

To produce lentivirus expressing miR-486a-5p, primary-miR-486a-5p sequence (Gene ID: 723876), which is comprised of a stem loop structure and 200 base pairs of upstream and downstream flanking genomic sequence, was inserted into the Lenti-miRs plasmid (Cat#MMIRXXX-PA-1, SBI, CA, USA). A scrambled negative control vector (Cat#MMIR000-PA-1, SBI, CA, USA) was used for producing control lentiviruses (Scramble). Lentivirus packaging was performed using a pPAKH1 Lentivirus Vector Packaging Kit (Cat#LV500A-1, System Biosciences, SBI, CA, USA) according to manufacturer's instructions. Briefly, the expression vector and package vectors were transfected into 293T cells using lipofectamine 3000 (Cat#L3000015, Invitrogen, Carlsbad, CA, USA) to generate the lentiviruses. After 36 hours, supernatants containing the lentiviruses (Lenti-miR-486-5p or Scramble) were harvested and the remaining cells were removed by filtration with 0.45 μm filters. After ultracentrifugation at 4000Xg at 4 °C for 5min, the lentiviruses were concentrated using PEG-it Virus Precipitation Solution (Cat#LV825A-1, SBI, CA, USA) according to the manufacturer's instructions.

### Mice

*S100a4-Cre* mice (C57BL/6JNju, N9) were acquired as a kind gift from Dr. Yu Nie at Fu Wai Hospital. S100 calcium binding protein A4 (*S100a4*), also known as fibroblast-specific protein-1 (FSP1), is expressed exclusively in fibroblast cells. The *S100a4-Cre* mouse line expresses Cre recombinase driven by the mouse *S100a4* promoter, and it has been previously used for the generation of cardiac fibroblast-specific gene knockout mice 28, 29. We crossbred *Fcer1a^flox/flox^* mice (C57BL/6, N9) with *S100a4-Cre* mice to generate *Fcer1a^flox/-^Cre^+/-^* mice. *Fcer1a^flox/-^Cre^+/-^* mice were crossbred with *Fcer1a^flox/flox^* mice to generate fibroblast FcεR1 conditional knockout mice *Fcer1a^flox/flox^Cre^+/-^* (FcεR1-cKO) and *Fcer1a^flox/flox^* control mice (FcεR1-Flox). All mice used in this study were littermates and syngeneic in the C57BL/6 background. To induce cardiac remodeling, 8-week-old male mice were implanted with osmotic pumps (Alzet MODEL 2002; DURECT, Cupertino, CA) that released Ang II (1000 ng/kg/min) for 14 days. To evaluate the therapeutic effect of miR-486a-5p on cardiac remodeling, lenti-miR-486a-5p (lenti-miR486) or scramble virus (1×10^7^ pfu viruses per mouse) was injected into mice through the tail vein on days 1 and 7 after Ang II infusion. Animals were handled in accordance with animal welfare regulations of the Peking Union Medical College, (Beijing, China) and our protocol was approved by the Animal Subjects Committee of Peking Union Medical College.

### Genotypic identification

FcεR1-cKO mice were identified using polymerase chain reaction (PCR) assays. Tail and heart tissues were obtained from 2-week-old mice, and genomic DNA was extracted with a Genomic DNA kit (TIANGEN DP304, China). Cre-mediated gene deletions were identified with WT primers: 5'F: CTAGGCCACAGAATTGAAAGATCT/3'R:GTAGGTGGAAATTCTAGCATCATCC and mutant primers: 5'F:GCGGTCTGGCAGTAAAAACTATC/3'R: GTGAAACAGCATTGCTGTCACTT. *Loxp* alleles were identified with the primers: 5'F: TCTGTCTAGGCCTTTCACAAGCAT,5'R:AGGATTTCAGGAGCAAGGAGATG,3'F:GAAAGTGGTTTGGTAAGCTGAAGG,3'R:CTGTGCTCTCTAGACCACTGTAGAG. For PCR, 2X Taq plus mix (TIANGEN, China) was used. For *loxp* allele identification, PCR conditions were: one cycle of 94 °C for 2 min; 35 cycles of 94 °C for 30 seconds (s), 60 °C for 30 s, and 72 °C for 30 s; and one cycle of 72 °C for 5 min. To identify cre-mediated genes, PCR conditions were: one cycle of 94 °C for 3 min; 35 cycles of 94 °C for 30 s, 52 °C for 1 min, and 72 °C for 1 min; one cycle of 72 °C for 2 min.

### Echocardiographic analysis

Mice cardiac function was evaluated by M-mode echocardiography on the short axis using a 30-MHz probe (Vevo 2100 system; Visual Sonics, Toronto, CA)^14^. The LV measurements were taken at the papillary muscle level. The left ventricle internal dimension (LVID), left ventricular posterior wall (LVPW), and left ventricular anterior wall (LVAW) were measured in systole and diastole. The LV ejection fraction (EF) and LV Fractional shortening (FS) were calculated. FS was calculated using the following formula: FS (%) = (LV end diastolic diameter - LV end systolic diameter)/LV end diastolic diameter*100%.

### Measurement of blood pressure by tail-cuff plethysmography

Systolic and diastolic blood pressures were measured using the non-invasive tail-cuff method (CODA, Kent Scientific, USA) as described previously 14. Briefly, mice were encouraged to walk into the restraint tubes and their tails were restrained from passing through the occlusion tail cuff. Then, the mice were warmed on the restraint platform heating pad at 37 ºC for about 5 min. Next, the mice were trained for 3-15 min until a stable blood pressure was recorded. Reported blood pressures were the average of at least five successful measurements.

### Tissue preparation and histological measurements

After 14 days of Ang II infusion, mouse hearts were harvested. To evaluate fibrotic areas and detect the fibrillary collagen, Sirius Red and Masson staining (Solarbio, Beijing, China) were performed as described previously 30. For immunohistochemical staining, primary antibodies and dilutions used were rabbit anti-Periostin antibody (Cat#ab215199, Abcam, 1:200), rabbit anti-α-SMA (Cat#ab5694, Abcam, 1:500), rabbit anti-Collagen Ⅰ (Cat#PA1-26204, Invitrogen, 1:200), and rabbit anti-Collagen Ⅲ (Cat#BA0326, Boster Biological Technology Co. Ltd, 1:200). The secondary antibody was HRP-labeled anti-rabbit IgG (Cat#ZDR-5306, Beijing Zsbio biotechnology, China). Aminoethyl carbazole (AEC) substrate kits (Cat#ZLI-9036, Beijing Zsbio biotechnology, China) were used for immunohistochemical staining. Images were acquired with Nikon microscopes. To quantitatively analyze fibrotic tissue area relative to the total heart tissue, a total of nine fields per mouse (three sections per mouse and three fields per section) were randomly selected, and statistical analysis was performed using Image-Pro Plus Software (Media Cybernetics, Bethesda, MD).

### Patient study

HF patients and age- and sex-matched healthy subjects (healthy physical examinees) were enrolled at Beijing Hospital and Teda International Cardiovascular Hospital from January 2019 to December 2020. HF patients were selected according to the following standards: (1) current or previous symptoms (limitation of activity, and dyspnea or orthopnea) and signs (edema, elevated jugular venous pressure, rales, or a third heart sound/gallop rhythm); (2) serum BNP ≥100 pg/mL and diagnosed clinical HF; (3) left ventricular ejection fraction (LVEF) ≤40%; (4) cardiac function that had remained stable over the previous six months; and (5) no changes in medication during the previous six months. HF patients were excluded if they had: (1) significant concomitant disease, such as pulmonary inflammation, renal failure, immune disease, infectious diseases, allergic diseases or cancer; (2) evidence of myocardial infarction or unstable angina during the previous six months; or (3) surgical history during the previous six months. Healthy subjects were collected from volunteers who received regular physical examinations in the healthcare center at the Beijing hospital. The Institutional Ethics Committee of Peking Union Medical College approved the investigation.

### Statistical analysis

All statistical analyses were performed using GraphPad Prism 8.0. Data are presented as mean ± SD in cell experiments and mean ± SEM in animal studies. First, the normality test was performed. Then, if the data were normally distributed, the Student's *t*-test was used to analyze the difference between two groups. If the data were not normally distributed, then the Mann-Whitney U test was chosen to test the difference between two groups. One-way or two-way ANOVA with the Bonferroni's post hoc test was performed to compare the differences among more than two groups. *P* < 0.05 was considered statistically significant.

## Results

### IgE promoted CF trans-differentiation and collagen expression via FcεR1

**Figure 1:**
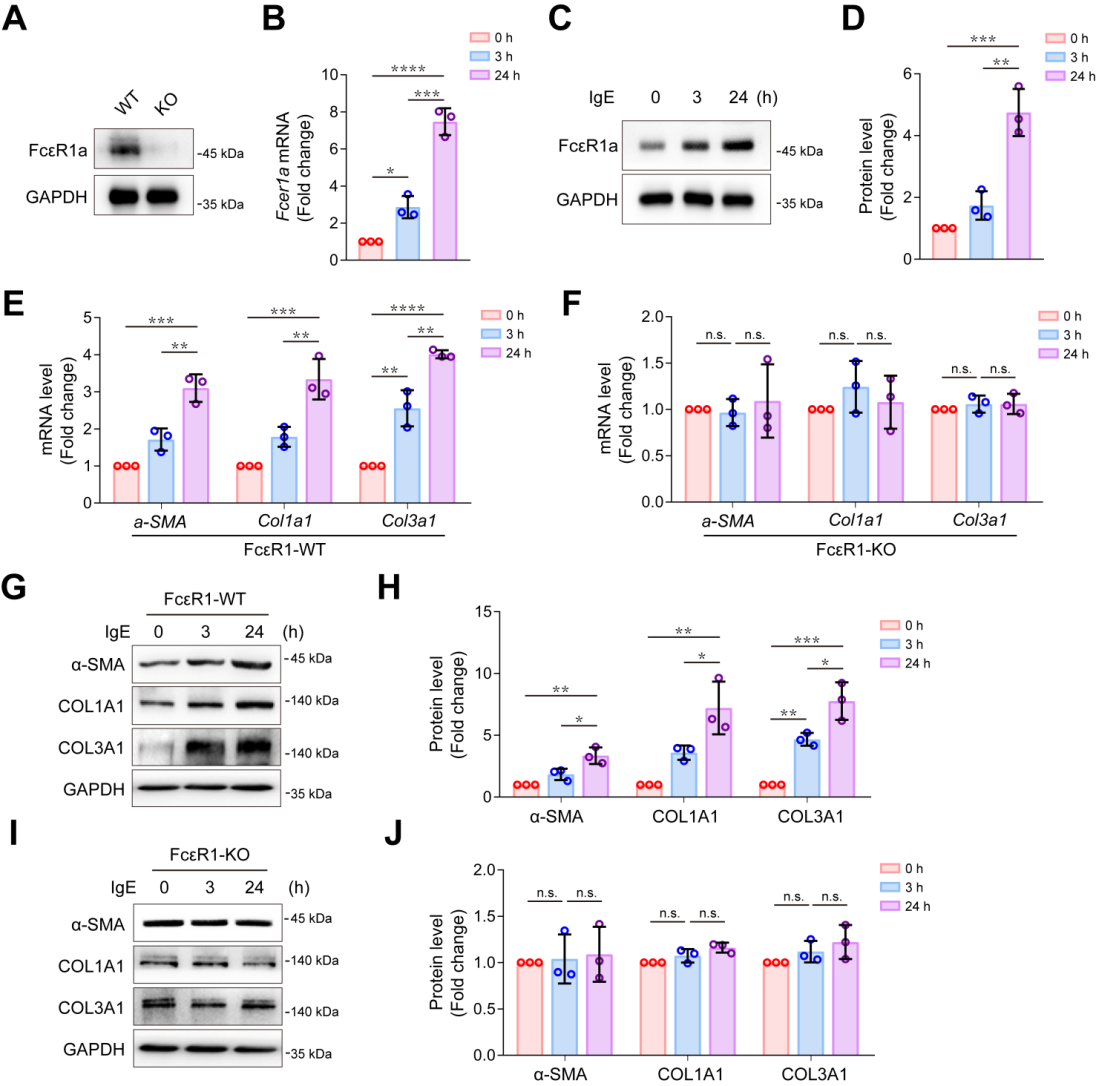
** Effects of IgE-FcεR1 signaling on CFs. (A)** FcεR1a expression levels in CFs of WT and FcεR1-KO mice measured by immunoblot. **(B-D)** FcεR1a expression levels after IgE treatment (5 μg/mL) for 0, 3, and 24 h measured by qPCR **(B)** and immunoblot **(C-D)**. **(E-F)** mRNA expression of key fibrotic genes (*a-SMA*, *Col1a1* and *Col3a1*) in IgE-stimulated FcεR1-WT **(E)** and FcεR1-KO **(F)** CFs at 0, 3, and 24 h. **(G)** Representative western blot of α-SMA, COL1A1, and COL3A1 in FcεR1-WT CFs after IgE treatment for 0, 3, and 24 h. **(H)** Statistical analysis of α-SMA, COL1A1, and COL3A1 protein expression in FcεR1-WT CFs, normalized to GAPDH (fold change versus 0 h). **(I)** Representative western blot of α-SMA, COL1A1, and COL3A1 in FcεR1-KO CFs after IgE treatment. **(J)** Statistical analysis of the relative protein expression of α-SMA, COL1A1, and COL3A1 in FcεR1-KO CFs. Data are mean ± SD from 3 independent experiments. *p < 0.05, **p < 0.01, ***p < 0.001, ****p < 0.0001, n.s indicates no significant difference in *One-way ANOVA* with Bonferroni's post hoc test.

We successfully isolated highly purified primary mouse CFs (Figure S1) and found that the IgE receptor, FcεR1, was expressed in CFs of WT mice but not in FcεR1-KO mice (Figure 1A). We stimulated CFs with IgE for different lengths of time (0, 3, and 24 h) and found that the expression of FcεR1 was increased in a time-dependent manner (Figure 1B-D and Figure S2A). Moreover, after IgE stimulation, we observed increased mRNA and protein expression of myofibroblast marker α-SMA, collagen Ⅰ and collagen III in WT CFs. The expression was time-dependent, and no significant changes were observed in FcεR1-KO CFs (Figure 1E-J and Figure S2B-E). These findings are consistent with our previous results with primary neonatal rat CFs 14.

### Fibroblast-specific depletion of FcεR1 alleviated Ang II-induced cardiac fibrosis

We previously showed that cardiac fibrosis was significantly reduced in global FcεR1 KO mice. To determine the direct contribution of CF-FcεR1 in cardiac fibrosis, we generated CF-specific FcεR1 KO mice (FcεR1-cKO). FcεR1-cKO mice were produced using an *S100a4-Cre* mouse line that expresses Cre recombinase exclusively in fibroblast cells. In brief, *Fcer1a^flox/-^ S100a4 Cre^+/-^* mice and *Fcer1a^flox/flox^* mice were crossbred to generate fibroblast FcεR1 conditional knockout mice *Fcer1a^flox/flox^Cre^+/-^* (FcεR1-cKO) and *Fcer1a^flox/flox^* control mice (FcεR1-Flox) (Figure S3A-B). To induce cardiac fibrosis, we infused FcεR1-Flox or FcεR1-cKO mice with saline or Ang II for two weeks. No blood pressure differences were observed between FcεR1-Flox and FcεR1-cKO mice (Figure S4A). We found that the average serum IgE levels of Ang II-infused mice were significantly increased compared with control groups (Figure S4B). Compared with controls, Ang II significantly increased the ratio of left ventricular weight to body weight in FcεR1-Flox mice (Figure S4C), which was somewhat attenuated in FcεR1-cKO mice (Figure S4C). Echocardiographic analysis showed that FcεR1 depletion alleviated Ang II-induced cardiac remodeling, as reflected by lower left ventricular mass and thinner diastolic (LVAW;d) and systolic (LVAW;s) left ventricular anterior wall thicknesses (Table S2).

**Figure 2:**
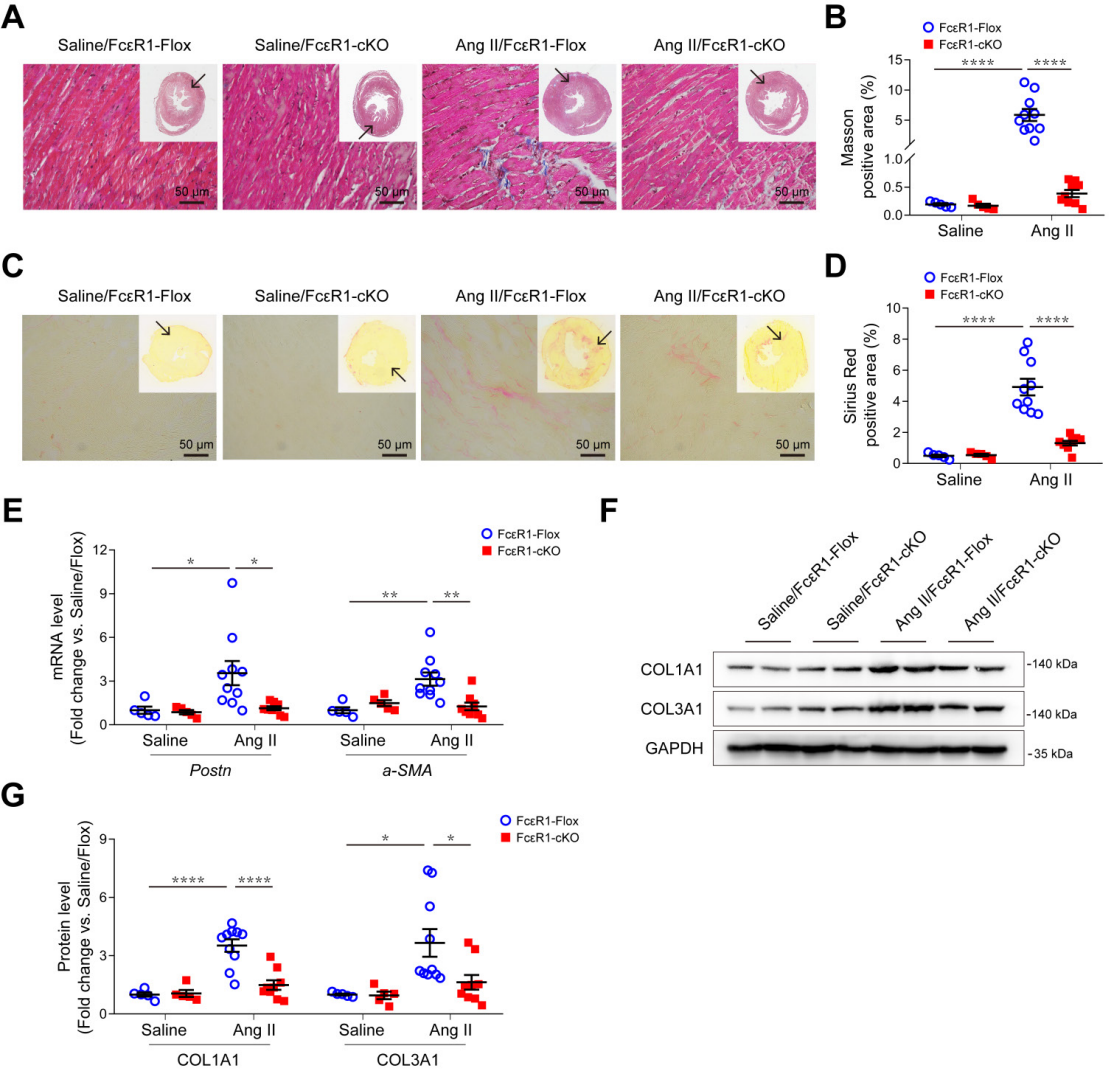
** Effects of CF FcεR1 deletion on Ang II-induced cardiac fibrosis. (A)** Representative images of Masson staining of the heart tissues from Ang II- or saline-infused FcεR1-Flox and FcεR1-cKO mice. Images were taken at 200X magnification. Scale bars, 50 μm. **(B)** Quantification of myocardial interstitial fibrosis by Masson staining. A total of nine fields from three sections (three fields from each section) per mouse were randomly selected for analysis. **(C)** Representative images of Sirius Red staining of the heart tissues from Ang II- or saline-infused FcεR1-Flox and FcεR1-cKO mice. Images were taken at 400X magnification. Scale bars, 50 μm. **(D)** Quantification of myocardial interstitial fibrosis by Sirius Red staining. A total of nine fields from three sections (three fields from each section) per mouse were randomly selected for analysis. **(E)** qPCR analysis of periostin (*Postn*) and *a-SMA* expression in the heart tissues of the four indicated groups. **(F)** Representative immunoblot analysis showing the expression of COL1A1 and COL3A1 in the hearts of Ang II- or saline-infused FcεR1-Flox and FcεR1-cKO mice. **(G)** Statistical analysis of COL1A1 and COL3A1 expression normalized to GAPDH. Total n = 5 (Saline/FcεR1-Flox), n = 5 (Saline/cKO), n = 10 (Ang II/FcεR1-Flox), or n = 9 (Ang II/cKO) per group. Results are shown as mean ± SEM. *p < 0.05, **p < 0.01, ****p < 0.0001 by Two-way ANOVA with Bonferroni's post hoc test.

We next performed Masson and Sirius Red staining to evaluate myocardial fibrosis in heart tissues. The results showed that Ang II induced a significant increase in myocardial interstitial fibrosis, which was significantly alleviated in the hearts of Ang II-infused FcεR1-cKO mice (Figure 2A-D and Figure S4D-E). Consistent with this, the expression of CF trans-differentiation markers (Periostin and α-SMA) were reduced in FcεR1-cKO hearts as assessed by qPCR (Figure 2E) and immunostaining (Figure S5A-D). In addition, immunoblot and immunostaining analysis showed that the expression of collagen Ⅰ and collagen Ⅲ were significantly lower in FcεR1-cKO than FcεR1-Flox mice (Figure 2F-G and Figure S5E-H). Together, these findings suggested that IgE-FcεR1 signaling played a critical role in cardiac fibrosis in CFs.

### IgE down-regulated miR-486a-5p in CFs

**Figure 3:**
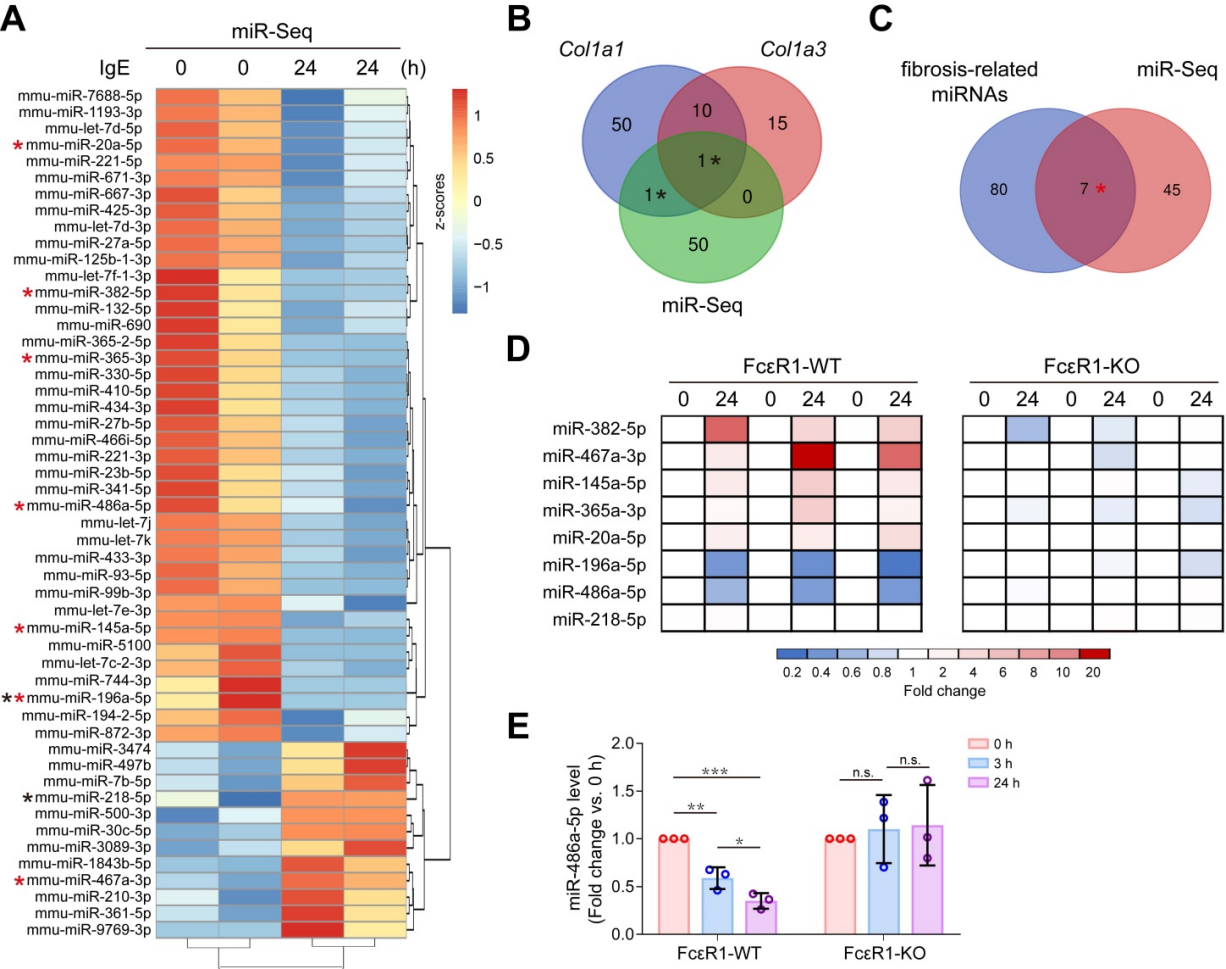
** IgE alters miRNAs profile in CFs and down-regulates miR-486a-5p. (A)** Heatmap illustrates differentially expressed miRNAs. The expression is normalized as row z-scores, up-regulation is indicated in red and down-regulation is indicated in blue. Each row represents a miRNA. **(B)** Three-way Venn diagram indicating the total number of miRNAs predicted to directly target *Col1a1* and *Col3a1*. The numbers of shared miRNAs are indicated in the intersections of the Venn diagram. Two miRNAs in the intersections are shown as black stars in (A). **(C)** Two-way Venn diagram indicating the miRNAs that may be involved in fibrosis. Seven miRNAs in the intersection are shown as red stars in (A). **(D)** qPCR analysis of eight candidate miRNAs from the intersections shown in (B-C). The expression levels of miRNAs in FcεR1-WT (left panel) and FcεR1-KO (right panel) CFs after IgE stimulation were normalized to U6. Three independent experiments were performed.** (E)** Expression of miR-486a-5p in IgE-stimulated FcεR1-WT and FcεR1-KO CFs at 0, 3, and 24 h measured by qPCR (fold change versus 0 h) in three independent experiments. Results are shown as mean ± SD. *p < 0.05, **p < 0.01, ***p < 0.001, n.s indicates no significance in *One-way ANOVA* with Bonferroni's post hoc test.

To explore if IgE contributes to cardiac fibrosis by regulating the miRNA profile, miRNA-seq for CFs treated with or without IgE was performed. The heatmap shows 52 differentially expressed miRNAs that responded to IgE treatment (Figure 3A). Among them, we found two candidate miRNAs (miR-196a-5p and miR-218-5p) that might directly target *Col1a1* or *Col3a1*, as indicated by the TargetScan7.1 database (Figure 3B). Further, to collect the IgE-sensitive miRNAs that may contribute to fibrosis by indirectly targeting collagen genes, we compared previously reported fibrosis-related miRNAs (literature reported fibrosis related miRNAs 15, 27 and fibrosis-related miRNA array results) with our miRNA-seq results. As shown in Figure 3C, seven miRNAs (miR-382-5p, miR-467a-3p, miR-145a-5p, miR-365-3p, miR-20a-5p, miR-196a-5p, and miR-486a-5p) were identified. To test if these eight bioinformatically suggested candidate miRNAs (miR-196a-5p, miR-218-5p, miR-382-5p, miR-467a-3p, miR-145a-5p, miR-365-3p, miR-20a-5p, and miR-486a-5p) are regulated by IgE-FcεR1 signaling, we evaluated their expression in FcεR1-WT (Figure 3D, left panel) and FcεR1-KO CFs (Figure 3D, right panel) using qPCR. Specifically, in IgE-treated FcεR1-WT CFs, miR-467a-3p expression was significantly increased and miR-196a-5p and miR-486a-5p expression was significantly decreased, while these changes were not observed in FcεR1-KO CFs (Figure 3D). The results were consistent with miRNA-Seq data. Because miR-486a-5p showed the highest basal expression level among these three candidates (Figure S6), we focused on this miRNA in our subsequent analysis. We stimulated FcεR1-WT and FcεR1-KO CFs with IgE for different lengths of time (0, 3, and 24 h), finding miR-486a-5p expression decreased in FcεR1-WT CFs in a time-dependent manner, but remained unchanged in FcεR1-KO CFs (Figure 3E). Taken together, these findings suggested that miR-486a-5p might play a role in the regulation of cardiac fibrosis by IgE-FcεR1.

### MiR-486a-5p directly regulated Smad1 in CFs

**Figure 4:**
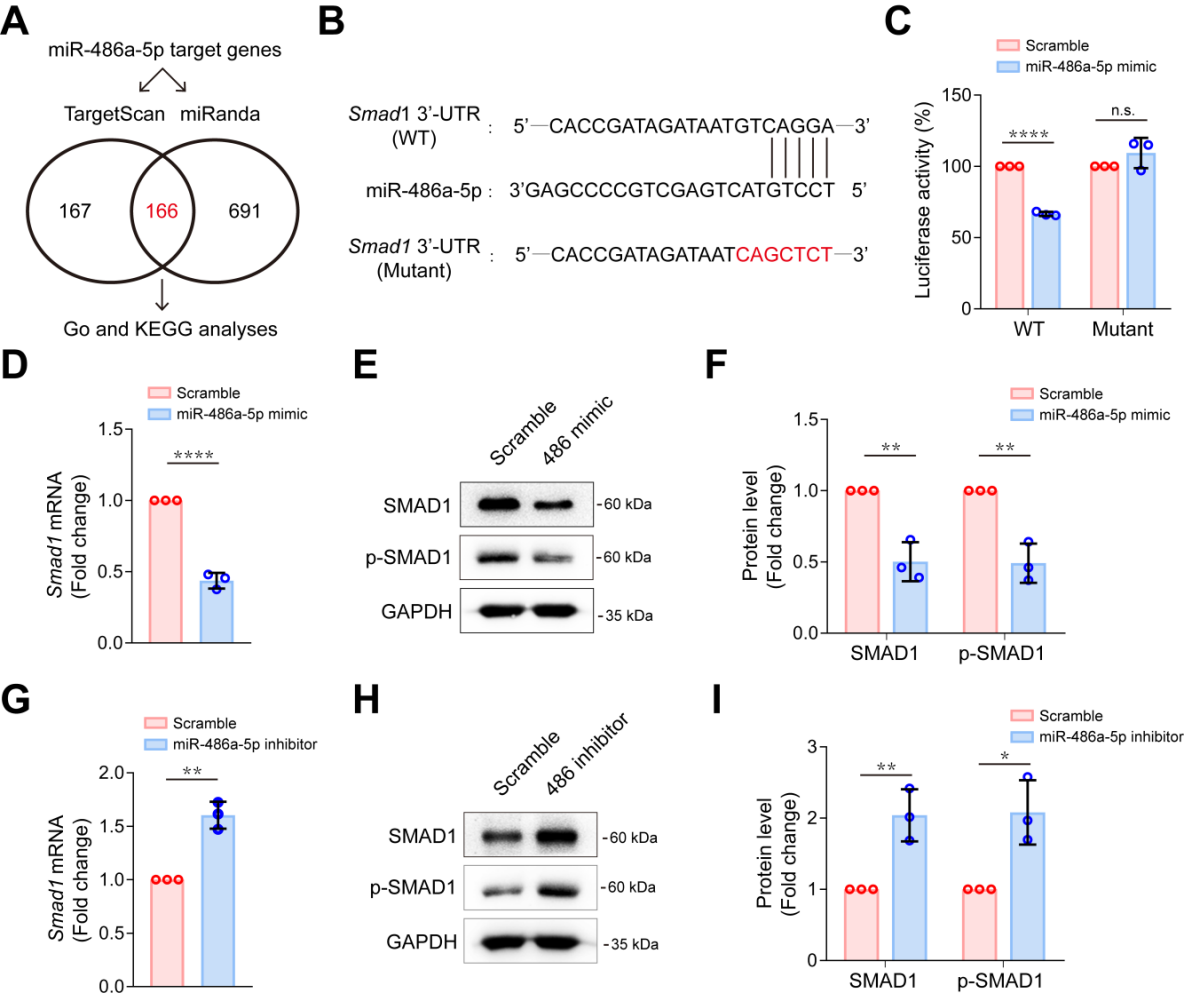
** MiR-486a-5p directly regulates *Smad1* in CFs. (A)** Potential miR-486a-5p targets from Targetscan7.1 and miRanda databases.** (B)** Diagram showing the predicted miR-486a-5p binding site on *Smad1* 3' UTRs, the sequence marked in red shows a mutation of the miR-486a-5p matched sequence.** (C)** Activity of the *Smad1* 3' UTR luciferase reporter was measured 24 h after transfection of miR-485a-5p mimic or scrambled controls in 293T cells. Renilla luciferase was used as the internal control. Three independent experiments were performed. **(D)** The expression of *Smad1* mRNA was reduced by transfection the miR-485a-5p mimic into CFs. **(E-F)** Representative immunoblot **(E)** and quantification **(F)** of SMAD1 and phospho-SMAD1 expression after miR-486a-5p overexpression in CFs. **(G)** The expression of *Smad1* mRNA was increased by transfection of the of miR-485a-5p inhibitor into CFs. **(H-I)** Representative immunoblot **(H)** and quantification **(I)** of SMAD1 and phospho-SMAD1 expression after miR-486a-5p knockdown in CFs. GAPDH was used for normalization. Quantification results are shown as mean ± SD. *p < 0.05, **p < 0.01, ****p < 0.0001, n.s indicates no significant difference using Student's *t*-test.

To explore the possible mechanism of miR-486a-5p participation in IgE-induced cardiac fibrosis, we predicted target genes for miR-486a-5p using combined Targetscan 7.1 and miRanda analyses, identifying 166 candidate target genes (Figure 4A, Table S3). To gain insights into the biological functions of the miR-486a-5p target genes, we performed GO analysis of these predicted genes and found 10 genes (*Foxo1*, *Smad1*, *Smad2*, *Eda2r*, *Igf1r*, *Il1a*, *Map3k7*, *Mapk8ip1*, *Mdfic*, and *Men1*) that appeared with high frequency (more than four times) in top 15 GO terms of annotated function (Figure S7A, Table S4). Further, KEGG analysis revealed top 10 KEGG pathways (Figure S7B). More importantly, five genes (*Smad1*, *Smad2*, *Igf1r*, *Sav1*, and *Tead1*) were predicted to be involved in at least two of these ten pathways (Figure S7B, Table S5). Three genes (*Smad1*, *Smad2* and *Igf1r*) were identified by overlapping the GO and KEGG results. *Smad1* and *Smad2* have both been implicated in tissue fibrosis 31, 32. Several studies 27, 33-35 have clearly demonstrated that *Smad2* is a direct target of miR-486a-5p and is negatively regulated by miR-486a-5p in fibroblasts. However, there have been fewer studies of *Smad1* 36. To test if *Smad1* is also a direct target of miR-486a-5p, dual-luciferase reporter assays were performed. We cloned wildtype (WT) and mutated *Smad1* 3'-UTR sequences into luciferase reporter plasmids (Figure 4B). As shown in Figure 4C, miR-486a-5p expression markedly inhibited the activity of WT reporters; however, the activity of mutated reporters remained unchanged. This confirms that *Smad1* was a novel direct target of miR-486a-5p. To investigate further, primary mouse CFs were transfected with miR-486a-5p mimics or inhibitor (Figure S8). As shown in Figure 4D-F, overexpression of miR-486a-5p in CFs suppressed the expression of SMAD1, and SMAD1 phosphorylation (p-SMAD1) was also decreased due to SMAD1 downregulation. In contrast, miR-486a-5p inhibition significantly increased the expression and phosphorylation of SMAD1 (Figure 4G-I). In addition, our data also confirmed that *Smad2* can be regulated by miR-486a-5p in CFs (Figure S9). Taken together, these results indicated that *Smad1* was a novel direct target of miR-486a-5p and was negatively regulated by miR-486a-5p in CFs, suggesting a role for *Smad1* in cardiac fibrosis induced by IgE/miR-486a-5p.

### MiR-486a-5p/Smad1 axis participated in IgE-induced collagen expression

**Figure 5:**
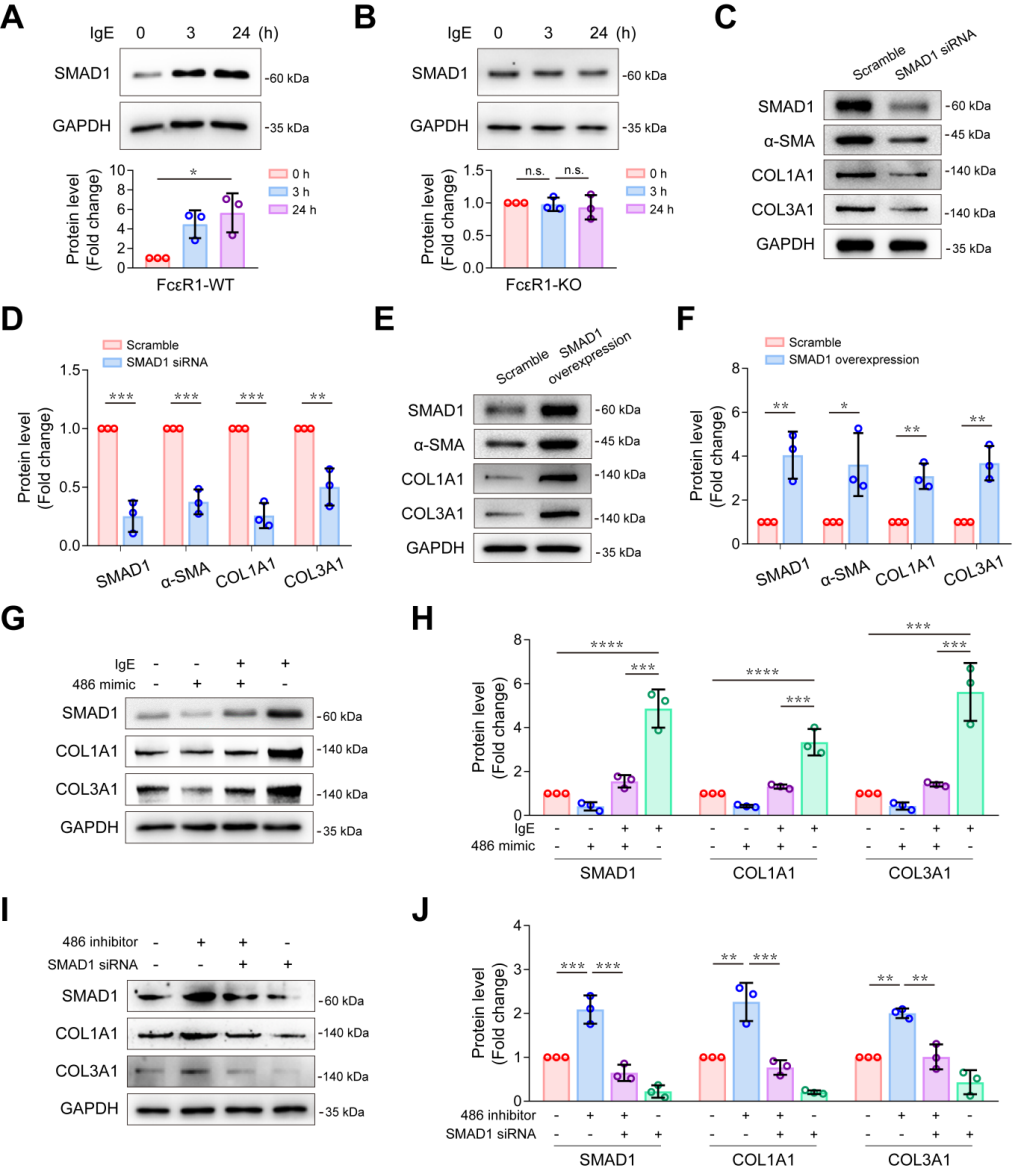
** IgE promotes collagen expression via miR-486a-5p/*Smad1* in CFs. (A-B)** Representative immunoblot (upper panel) and quantification (lower panel) of SMAD1 protein expression in IgE-stimulated WT **(A)** and FcεR1-KO **(B)** CFs at indicated lengths of time (0, 3, 24 h). **(C-D)** Representative immunoblot **(C)** and quantification **(D)** showing protein levels of α-SMA, COL1A1, and COL3A1 in CFs transfected with small interference RNA (siRNA) against Smad1 or scrambled control. **(E-F)** Representative immunoblot **(E)** and quantification **(F)** showing protein levels of α-SMA, COL1A1, and COL3A1 in CFs transfected with *Smad1* ORF clone or scrambled control. **(G-H)** Representative immunoblot **(G)** and quantification **(H)** of SMAD1, COL1A1, and COL3A1 protein expression in CFs transfected with miR-486a-5p mimic and/or IgE. **(I-J)** Immunoblot **(I)** and quantification **(J)** showing protein levels of SMAD1, COL1A1, and COL3A1 in CFs transfected with siRNA against *Smad1* and/or miR-486a-5p inhibitor. Data are mean ± SD from 3 independent experiments. *p < 0.05, **p < 0.01, ***p < 0.001, ****p < 0.0001, n.s indicates no significant difference in *One-way ANOVA* with Bonferroni's post hoc test **(A-B)**, Student's *t*-test **(D, F)** or *Two-way ANOVA* with Bonferroni's post hoc test **(H, J)**.

To explore whether *Smad1* plays a role in IgE-induced fibrosis, we first evaluated the effect of IgE on *Smad1* expression. We treated FcεR1-WT and FcεR1-KO CFs with IgE for 0, 3, and 24 hours. As shown in Figure S10 and Figure 5A-B, IgE significantly upregulated both mRNA and protein levels of *Smad1* expression in FcεR1-WT CFs, but not in FcεR1-KO CFs, suggesting IgE upregulates *Smad1* expression via FcεR1 in CFs. To further determine the role of *Smad1* in collagen expression, FcεR1-WT CFs were treated with *Smad1* siRNA or scrambled siRNA control for 24 h. Immunoblots showed that the expression of collagen Ⅰ and collagen Ⅲ as well as α-SMA were significantly reduced in *Smad1*-knockdown CFs (Figure 5C-D). In contrast, *Smad1* overexpression promoted CF activation and collagen expression (Figure 5E-F).

Therefore, we hypothesized that an miR-486a-5p/*Smad1* axis participated in IgE-induced fibrosis. To test this hypothesis, rescue assays were performed. First, to determine if IgE-induced collagen Ⅰ and collagen III expression is mediated by an miR-486a-5p pathway, we transfected CFs with miR-486a-5p mimic or scrambled control for 24 hours, followed by a 24-hour IgE treatment. As shown in Figure S11A, the expression of miR-486a-5p was increased after transfection of the miR-486a-5p mimic. IgE-induced upregulation of *Smad1* and fibrotic genes (*Col1a1* and *Col3a1*) was significantly abolished by miR-486a-5p overexpression (Figure S11B and Figure 5G-H). To determine if *Smad1* participates in the regulation of collagen expression by miR-486a-5p, we transfected CFs with siRNA against *Smad1*, followed by the miR-486a-5p inhibitor. The results showed that *Smad1* knockdown abrogated the upregulation of collagen Ⅰ and collagen Ⅲ caused by miR-486a-5p inhibition (Figure 5I-J). Taken together, these findings suggested that the miR-486a-5p/*Smad1* pathway mediated IgE-induced collagen expression in CFs.

### Overexpression of miR-486a-5p attenuated Ang II-induced cardiac fibrosis

**Figure 6:**
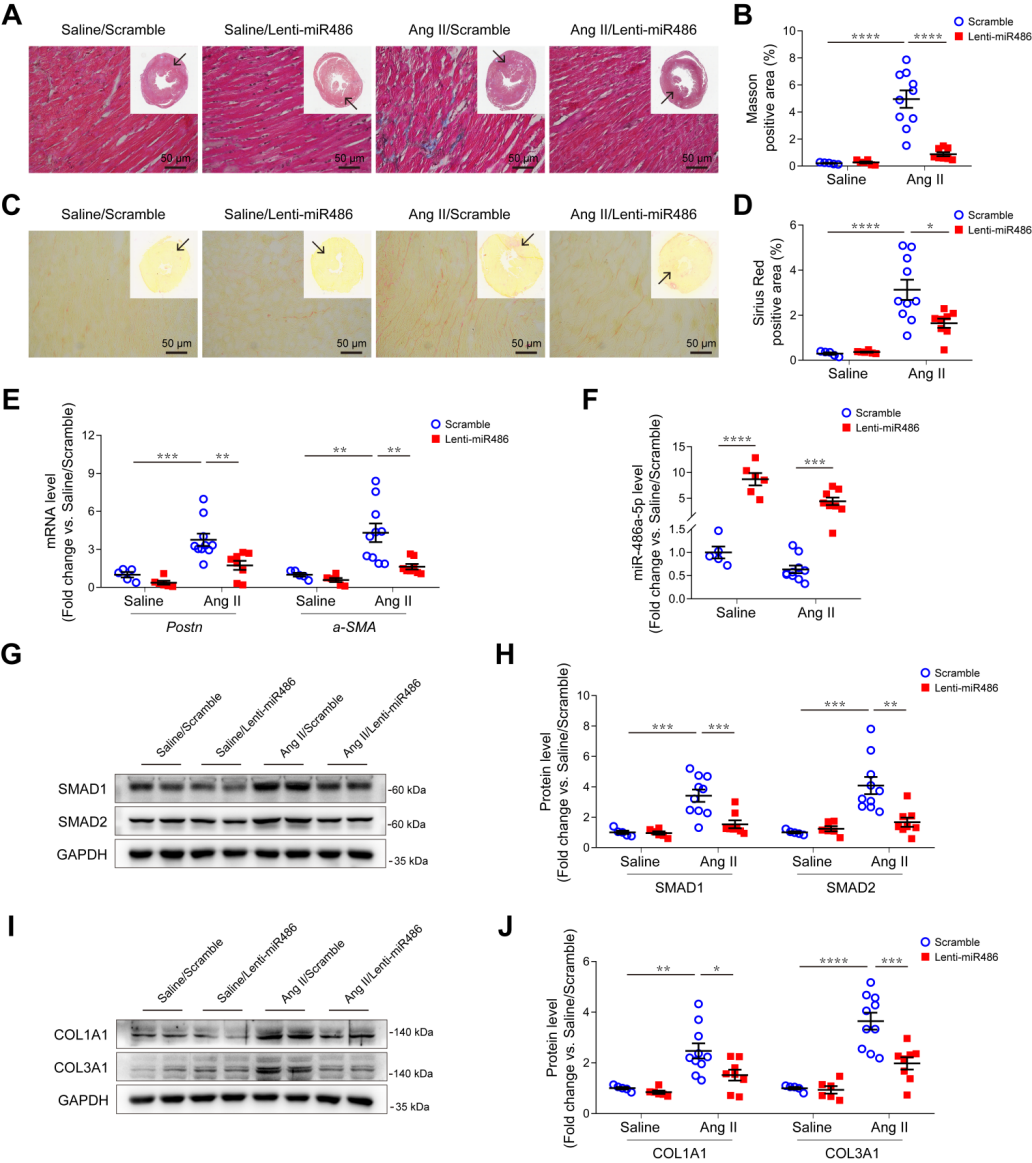
** Effects of miR-486a-5p overexpression on Ang II-induced cardiac fibrosis. (A)** Representative images of Masson staining of the heart tissues from lenti-miR486 or scramble-treated Ang II- or saline-infused mice. Images were taken at 200X magnification. Scale bars, 50 μm. **(B)** Quantification of myocardial interstitial fibrosis by Masson staining. A total of nine fields from three sections (three fields from each section) per mouse were randomly selected for analysis. **(C)** Representative images of Sirius Red staining of the heart tissues from lenti-miR486 or scramble-treated Ang II- or saline-infused mice. Images were taken at 400X magnification. Scale bars, 50 μm. **(D)** Quantification of myocardial interstitial fibrosis by Sirius Red staining. A total of nine fields from three sections (three fields from each section) per mouse were randomly selected for analysis. **(E)** qPCR analysis of *Postn* and *a-SMA* expression in the hearts of lenti-miR486 or scramble-treated Ang II- or saline-infused mice. **(F)** qPCR analysis of miR-486a-5p expression in the heart tissues of the 4 indicated groups. **(G-H)** Representative immunoblot **(G)** and quantification **(H)** showing the expression of SMAD1 and SMAD2 in the hearts of Ang II- or saline-infused mice treated with lenti-miR486 or scrambled control lentiviruses. **(I-J)** Immunoblot **(I)** and quantification **(J)** showing protein levels of COL1A1 and COL3A1 in the heart tissues from the indicated mice. Total n = 5 (Saline/Scramble), n = 6 (Saline/Lenti-miR486), n = 10 (Ang II/Scramble), or n = 8 (Ang II/Lenti-miR486) per group. Results are shown as mean ± SEM. *p < 0.05, **p < 0.01, ***p < 0.001, ****p < 0.0001 by *Two-way ANOVA* with Bonferroni's post hoc test.

To further explore the role of miR-486a-5p in cardiac fibrosis *in vivo*, we used a lentiviral transduction system to overexpress miR-486a-5p (lenti-miR486) or a lenti-scramble control (scramble) in mice. GFP-tagged lentiviruses were delivered by tail-vein injection at day 1 and 7 after saline or Ang II infusion. The overexpression of lentiviruses in heart tissues was confirmed by immunoblots and GFP immunofluorescent staining (Figure S12A-B). There were no blood pressure differences observed between lenti-miR486- and scramble-treated mice (Figure S13A). Ang II infusion increased left ventricular mass versus body weight, which was significantly abolished by miR-486a-5p overexpression (Figure S13B). Echocardiography measurements showed a significant decrease of LVAW;d, LVAW;s, and LV mass (Table S6), indicating miR-486a-5p overexpression alleviated Ang II-induced cardiac remodeling. Notably, Ang II-induced myocardial interstitial fibrosis was significantly alleviated by miR-486a-5p overexpression as indicated by Masson and Sirius Red staining (Figure 6A-D and Figure S13C-D). Consistent with this, the expression of fibrotic markers periostin and α-SMA, detected by qPCR (Figure 6E) and immunostaining (Figure S14A-D), were also significantly decreased in the hearts of lenti-miR486-treated mice compared with scramble-treated mice. Furthermore, we detected increased miR-486a-5p levels in the hearts of lenti-miR486-treated mice (Figure 6F) and found that miR-486a-5p overexpression downregulated Ang II-induced SMAD1, SMAD2, and collagen expression as assessed by immunoblots and immunostaining (Figure 6G-J and Figure S14E-H). Together, these data suggested that miR-486a-5p overexpression inhibited collagen expression and alleviated Ang II-induced cardiac fibrosis.

### Reduced serum miR-486-5p was correlated with HF markers in humans

**Figure 7:**
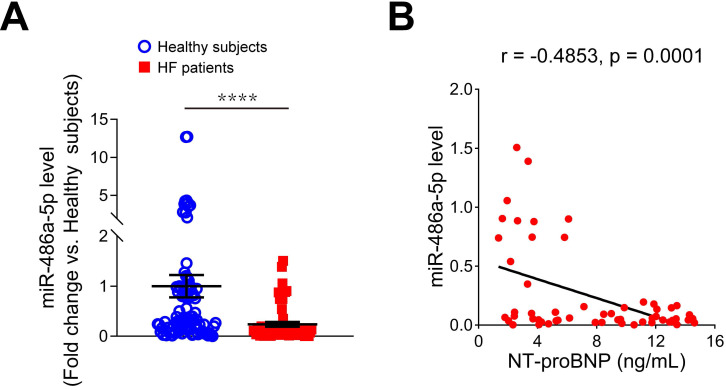
** Expression of miR-486-5p in the serum samples of HF patients. (A)** qPCR analysis of miR-486-5p expression in the serum samples of HF patients (n = 57) and age- and sex-matched healthy subjects (n = 87) (fold change versus healthy subjects). The results are shown as mean ± SEM. Statistical analysis were performed using the Mann-Whitney U test, ****p < 0.0001. **(B)** Pearson correlation analysis of the relationship between serum IgE and serum NT-proBNP in HF patients (n = 57).

To investigate the relationship between miR-486-5p and HF, we measured serum miR-486-5p levels of HF patients by qPCR and found miR-486-5p was significantly lower in the serum of HF patients (n = 57) than in age- and sex-matched healthy subjects (n = 87) (Figure 7A). To explore the clinical significance of reduced serum miR-486-5p levels, Pearson correlation tests were conducted between miR-486-5p and NT-proBNP (a biochemical marker of HF) levels in HF patients. Interestingly, the levels of serum miR-486-5p were negatively correlated with NT-proBNP (Figure 7B). These findings suggested that reduced serum miR-486-5p levels were correlated with HF in humans.

## Discussion

Our prior study demonstrated a role for IgE-FcεR1 in pathological cardiac remodeling and dysfunction 14. This study further clarifies the specific contribution of CFs in cardiac fibrosis mediated by IgE-FcεR1 *in vitro* and *in vivo*. Importantly, miR-seq and experimental validation revealed that miR-486a-5p was a crucial regulator of IgE-FcεR1-induced collagen expression in CFs, and *Smad1* participated in this process downstream of miR-486a-5p. We found that miR-486a-5p overexpression in mice alleviated Ang II-induced cardiac fibrosis. Serum miR-486-5p was also decreased in HF patients. Together, these findings suggested that this IgE-sensitive pathway played an important role in the pathogenesis of cardiac fibrosis. IgE protein exists in all mammals, and abnormal elevation of IgE could occur in diseases such as allergic diseases (asthma, food allergy) and IgE light chain diseases (macroglobulinemia, lymphoma, multiple myeloma). It is worth mentioning that HF is a common complication of light chain diseases, such as myeloma, due to the cardiotoxicity of chemotherapy 37, 38. Whether these diseases with abnormal IgE elevation contribute to cardiac fibrosis or not remains to be investigated, and our work has potential impacts on the treatment of patients with these diseases.

Consistent with our previous findings, after Ang II infusion serum IgE was significantly elevated in mice via as yet undetermined mechanisms. IgE is produced by activated IgE (+) B cells, and we found that Ang II infusion led to a marked increase in B cell activation in vasculature (data not shown). Thus, we speculate that B cell accumulation and activation in vasculature may lead to the production of IgE locally and in the circulatory system. Combined with our previous work, we confirmed that IgE-FcεR1 activation is one of the mechanisms of Ang II-mediated cardiac fibrosis *in vivo*, and our *in vitro* results rule out the possibility that Ang II can directly act on FcεR1 to promote fibrosis (Figure S15). We noticed that fibroblast-specific depletion of FcεR1 alleviated cardiac remodeling, so we performed wheat germ agglutinin (WGA) staining to evaluate cardiac hypertrophy. The results showed that CF FcεR1 depletion tended to alleviate Ang II-induced cardiomyocyte hypertrophy, but this effect did not reach statistical significance (Figure S16). We speculate that the changes of cardiomyocyte hypertrophy in cKO mice may be due to an intercellular interaction between cardiomyocytes and CFs, and the decreased heart weight may result from a reduction of myocardial collagen content as well as cardiomyocyte weight. We note that the concentration of IgE used in our *in vitro* studied experiments (5 μg/mL purified IgE) is higher than baseline serum IgE levels in mice and normal humans. Though, it is possible that IgE levels in the local microenvironment of a tissue *in vivo* may be much higher than the circulatory IgE levels, which should be tested in the future.

A growing list of miRNAs have been implicated as regulators of myocardial fibrosis 15. They act by targeting multiple fibrogenic cascades 39. For example, miR-15 has been shown to regulate cardiac fibrosis by targeting TGF-β signaling involving *Tgfb1*, *Mapk14*, *Eng*, *Smad3*, and *Smad7* 40. In CFs, miR-29 was suggested to repress ECM production by directly targeting *Col1a1*, *Col1a2*, *Col3a1*, *Mmp2*, *Eln*, *Fbn1* 41, 42. Here, we report that miR-486a-5p is a key mediator of IgE*-*FcεR1 signaling in CFs and has remarkable anti-fibrotic activity in myocardium *in vitro* and *in vivo*, consistent with previous studies that show miRNA‑486‑5p is involved in lung fibrosis and hypertrophic scar formation 27, 33. Although miR-486a-5p-overexpressing lentiviruses were successfully delivered to CFs in our study (Figure S12), the delivery was non-specific and the lentiviruses infected other cell types in the heart, such as cardiomyocytes. Thus, cell type-specific overexpression or knockdown of miR-486a-5p is needed to clarify the contributions of individual cell types. In addition, miR-486-5p has been reported to play a cardioprotective role in cardiomyocyte apoptosis caused by coronary microembolization or myocardial ischemia-reperfusion injury 43, 44, which further demonstrates the importance of miR-486a-5p as a protective factor in the heart.

How the IgE-FcεR1 pathway regulates miR-486a-5p is not clear. Because miRNAs are usually regulated by transcriptional factors (TFs), we used the TransmiR v2.0 database (http://www.cuilab.cn/transmir) to predict the upstream TFs of mmu-miR-486a and hsa-miR-486-1. Then, we overlapped these results with differentiated mRNAs in IgE-stimulated CFs identified in our previous RNA-seq study. We found that two predicted TFs (EZH2 and RAD21) were up-regulated in IgE-stimulated CFs. We think EZH2 and RAD21 may be part of the downstream signaling pathway of IgE-FcεR1, which regulates the expression of miR-486a-5p, but this hypothesis needs to be further verified. In addition, FcεR2, a low affinity IgE receptor, has been reported to regulate immune responses 45. The potential regulation of miR-486a-5p by IgE-FcεR2 in IgE-induced cardiac fibrosis warrants further investigation. We have previously confirmed that TGF-β is a crucial mediator in IgE-FcεR1-induced CF activation and cardiac fibrosis 14. Though we hypothesized miR-486a-5p could regulate TGF-β, we found no changes in TGF-β expression after overexpression or inhibition of miR-486a-5p in CFs (Figure S17), suggesting miR-486a-5p promotes fibrotic response via a TGF-β-independent mechanism.

In addition to miR-486a-5p, the other two candidate miRNAs (miR-467a-3p, miR-196a-5p) identified by our bioinformatic analysis may also be involved in IgE*-*FcεR1-mediated cardiac fibrosis. A previous study showed that miR-196a directly regulated *Col1a1* and *Col3a1* expression in keloid fibroblasts 46, and miR-196a/*Col1a1* has been reported to participate in pulmonary fibrosis 47, though its roles in CFs and cardiac fibrosis remain unknown. It has been reported that miR-467a-3p is nearly undetectable under basal conditions but highly upregulated in response to hyperglycemia in microvascular endothelial cells 48. Similarly, miR-467a-3p was poorly expressed in CFs and upregulated by IgE stimulation in our study. The potential involvement of miR-467a-3p in IgE-induced cardiac fibrosis needs to be studied further.

SMAD1 belongs to the SMAD family of proteins, which are critical for regulating cell development and growth. The role of SMAD1 signaling in fibrosis has not been conclusively determined 31. Some emerging evidence suggests that SMAD1 is implicated in fibrotic pathogenesis; for example, it was reported that SMAD1 promoted streptozotocin- and Ang II-induced diabetic mesangial matrix expansion by directly upregulating Type IV collagen synthesis 49-51. Schwartze *at al.* showed that SMAD1 is a critical regulator of lung fibroblast differentiation into myofibroblast in lung diseases treated with glucocorticoid 52. SMAD1 activation is also involved in liver fibrosis and systemic sclerosis 53-55. Consistent with this, our results show that *Smad1* is a novel target of miR-486a-5p and a regulator of collagen expression in CFs in response to IgE-FcεR1 signaling. We cannot exclude the possibility that other targets of miR-486a-5p, such as *Smad2* and *Igf1r*, may be involved in cardiac fibrosis. SMAD2 signaling is widely regarded as one of the principal fibrotic mediators that promotes cardiac fibrosis 32, 56, attributed a crucial role in miR-486a-5p-mediated anti-fibrotic effects in other tissues 27, 33. Our findings confirm that *Smad2* can be regulated by miR-486a-5p in CFs (Figure S9). In addition, although *Smad3* was not among the candidate targets of miR-486a-5p we predicted, SMAD3 is also considered an essential fibrotic factor 57. Whether *Smad3* can be indirectly regulated by miR-486a-5p remains unknown. Therefore, further research is needed to clarify the contributions of these various factors in miR-486a-5p-mediated regulation of cardiac fibrosis.

Extending previous research, this study further clarifies the specific contribution of CFs in IgE-induced fibrosis. We show that miR-486a-5p/*Smad1* signaling, in addition to the TGF-β pathway 14, is another important component of IgE-induced fibrotic response revealing a new potential target for the treatment of cardiac fibrosis.

## Abbreviations

Ang II: angiotensin II
CF: cardiac fibroblast
cKO: conditional knockout
ECM: extracellular matrix
IgE: immunoglobulin E
LVAW: the anterior left ventricular wall
LVPW: the posterior left ventricular wall
miRNA: microRNA
TGF-β1: transforming growth factors-β1
NT-proBNP: N-terminal pro-B-type natriuretic peptide
