# Supplementary material for: Elevated IgE promotes cardiac fibrosis by suppressing miR-486a-5p
Source: Theranostics. 2021 Jun 5;11(15):7600–15. doi: 10.7150/thno.47845 (PMC8210611; doi:10.7150/thno.47845)
Supplement: Supplementary file 1 — Supplementary figures and tables. [file thnov11p7600s1.pdf]

# SUPPLEMENTARY MATERIAL

**This file includes:**

**Supplementary Figures and Figure Legends**

Figure S1. The purity of the extracted CFs

Figure S2. Expression of FcεR1 and fibrotic genes after IgE treatment at different times and doses in CFs

Figure S3. Construction of FcεR1-cKO mice

Figure S4. Effect of CF FcεR1 deletion on Ang II-infused mice

Figure S5. Immunohistochemical staining of fibrotic markers in heart tissues from CF-specific FcεR1 KO mice

Figure S6. Basal levels of miRNAs in CFs

Figure S7. Gene ontology and KEGG Pathway enrichment analyses for screening potential miR-486a-5p targets

Figure S8. Efficiency data for miR-486a-5p mimic and miR-486a-5p inhibitor

Figure S9. Expression of SMAD2 and phospho-SMAD2 after miR-486a-5p overexpression or knockdown in CFs

Figure S10. SMAD1 mRNA expression after IgE stimulation in CFs

Figure S11. Rescue assays performed in CFs

Figure S12. Overexpression of lenti-miR486 indicated by GFP detection

Figure S13. Effect of miR-486a-5p overexpression on Ang II-infused mice

Figure S14. Immunohistochemical staining of fibrotic markers in heart tissues from miR-486a-5p-overexpressed mice

Figure S15. Effect of Ang II on WT and FcεR1-KO CFs *in vitro*

Figure S16. Effect of CF FcεR1 deletion on Ang II-induced cardiomyocyte hypertrophy

Figure S17. Expression of TGF-β after miR-486a-5p overexpression or knockdown in CFs

**Supplementary Tables**

Table S1. Primer sequences (provided as an Excel file)

Table S2. Echocardiographic analysis of Ang II- or saline-infused FcεR1-Flox and FcεR1-cKO mice

Table S3. Candidate miR-486a-5p targets predicted by Targetscan 7.1 and miRanda combined analyses (provided as an Excel file)

Table S4. GO analysis of the candidate miR-486a-5p targets (provided as an Excel file)

Table S5. KEGG analysis of the candidate miR-486a-5p targets (provided as an Excel file)

Table S6. Echocardiographic analysis of Ang II- or saline-infused WT mice treated with lenti-miR-486a-5p or scramble

## Supplementary Figures and Figure Legends

### Figure S1

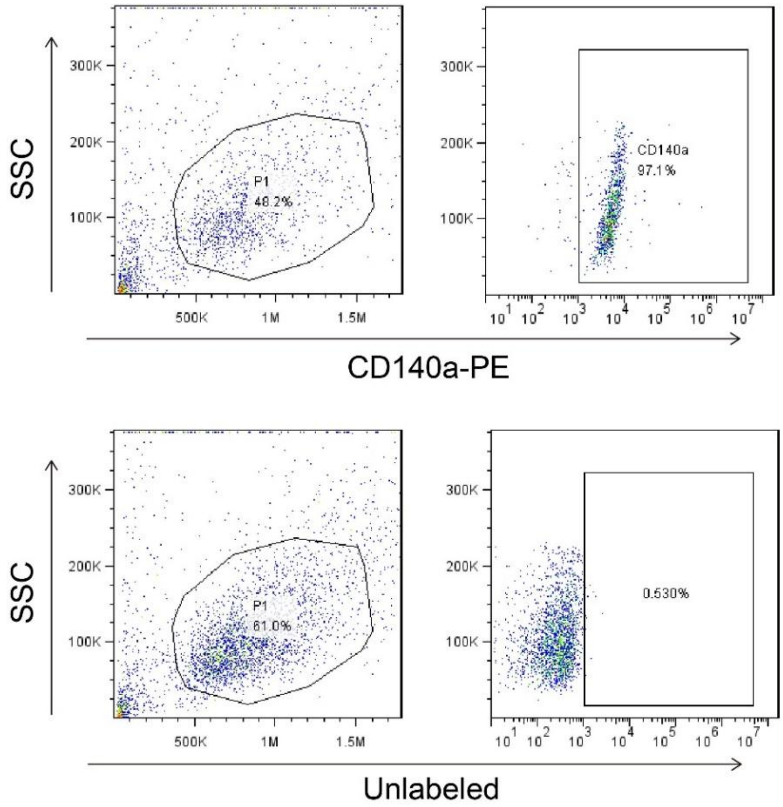


**Figure S1. The purity of the extracted CFs**

Flow cytometry analysis of PDGFR-α (CD140a) $^{+}$ cells in the isolated primary mouse CFs.

**Figure S2**

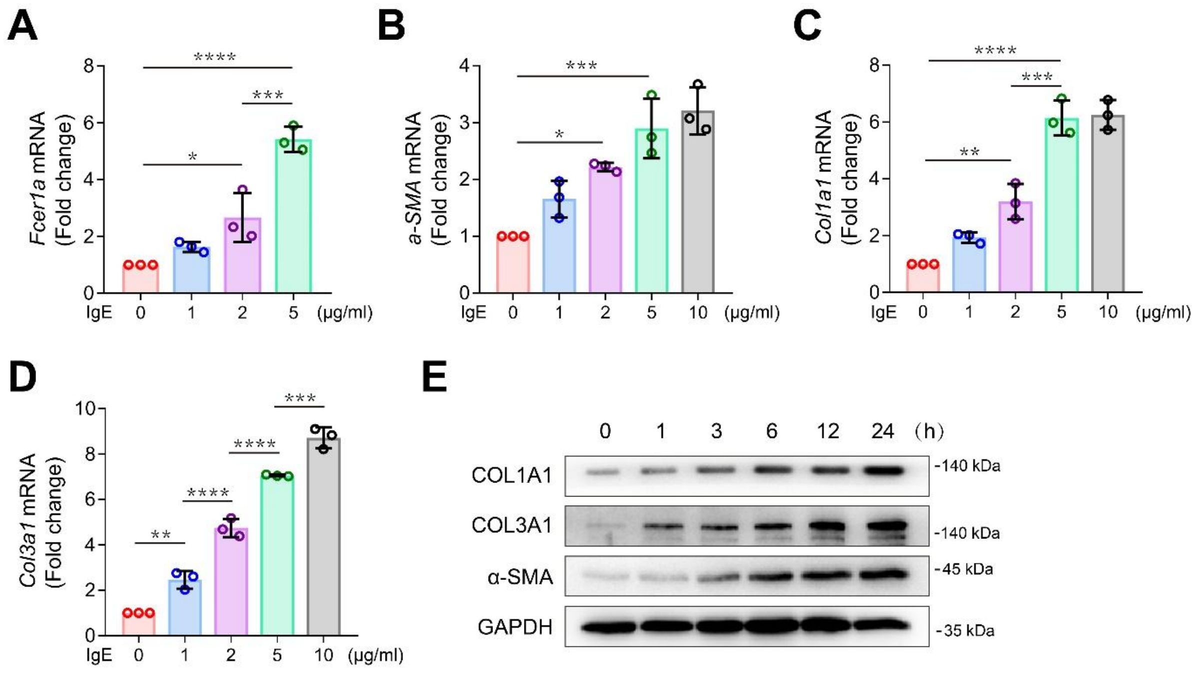


**Figure S2. Expression of FcεR1 and fibrotic genes after IgE treatment at different times and doses in CFs**

**A**. *Fcer1a* mRNA expression levels in mouse primary CFs after IgE stimulation at different concentrations (0, 1, 2, and 5 μg/ml). **B–D.** qPCR analysis of key fibrotic genes (*a-SMA*, *Col1a1*, and *Col3a1*) mRNA expression in 0, 1, 2, 5, and 10 μg/ml IgE-stimulated FcεR1-WT CFs. **E.** Immunoblot analysis of α-SMA, COL1A1, and COL3A1 protein expression after IgE treatment of CFs at 0, 1, 3, 6, 12, and 24 hours. Data are mean ± SD from 3 independent experiments. *$p < 0.05$, **$p < 0.01$, ***$p < 0.001$, ****$p < 0.0001$ by *one-way ANOVA* with Bonferroni's post hoc test.

**Figure S3**

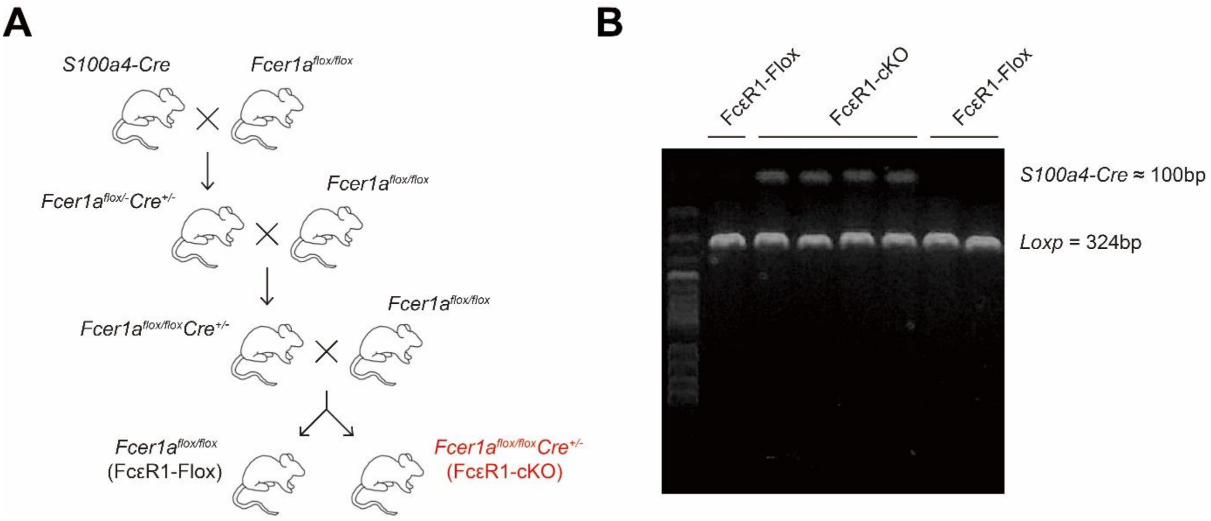


**Figure S3. Construction of FcεR1-cKO mice**

**A–B.** The construction pipeline **(A)** and identification **(B)** of FcεR1-cKO mice.

## Figure S4

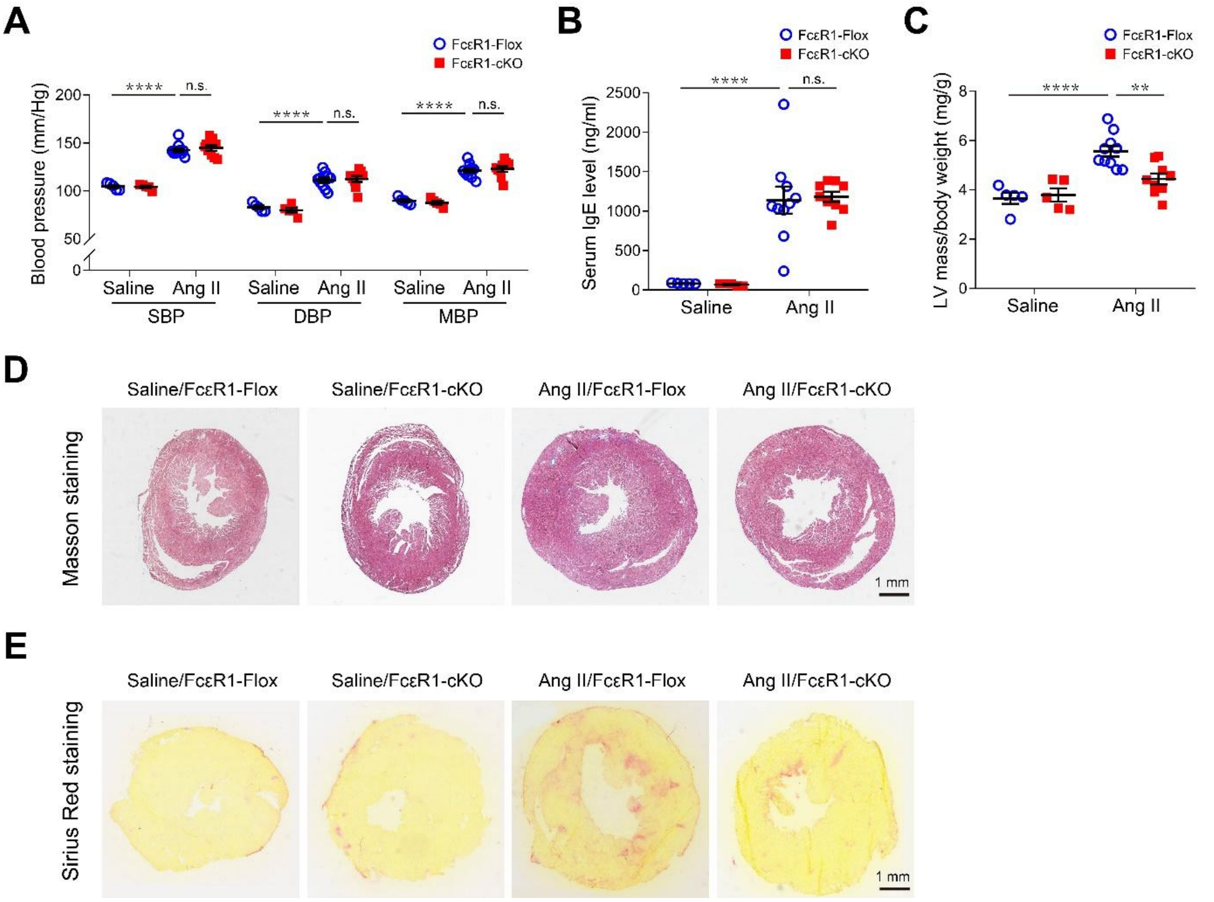


**Figure S4. Effect of CFs FcεR1 deletion on Ang II-infused mice**

**A.** Systolic blood pressure (SBP), diastolic blood pressure (DBP), and mean blood pressure (MBP) were measured by non-invasive tail-cuff monitor in Ang II- or saline-infused FcεR1-Flox and FcεR1-cKO mice. **B.** ELISA analysis of serum IgE in Ang II- or saline-infused FcεR1-Flox and FcεR1-cKO mice. **C.** Left ventricular weight versus body weight after 2-week Ang II treatment in FcεR1-Flox and FcεR1-cKO mice. **D-E.** Representative heart sections examined by Masson **(D)** and Sirius Red staining **(E)**. Scale bars, 1 mm. Total n = 5 (Saline/FcεR1-Flox), n = 5 (Saline/FcεR1-cKO), n = 10 (Ang II/FcεR1-Flox) or n = 9 (Ang II/FcεR1-cKO) per group. The results are shown as mean ± SEM. $^{**}p < 0.01$, $^{****}p < 0.0001$, n.s. indicates no significance in *Two-way ANOVA* with Bonferroni's post hoc test.

**Figure S5**

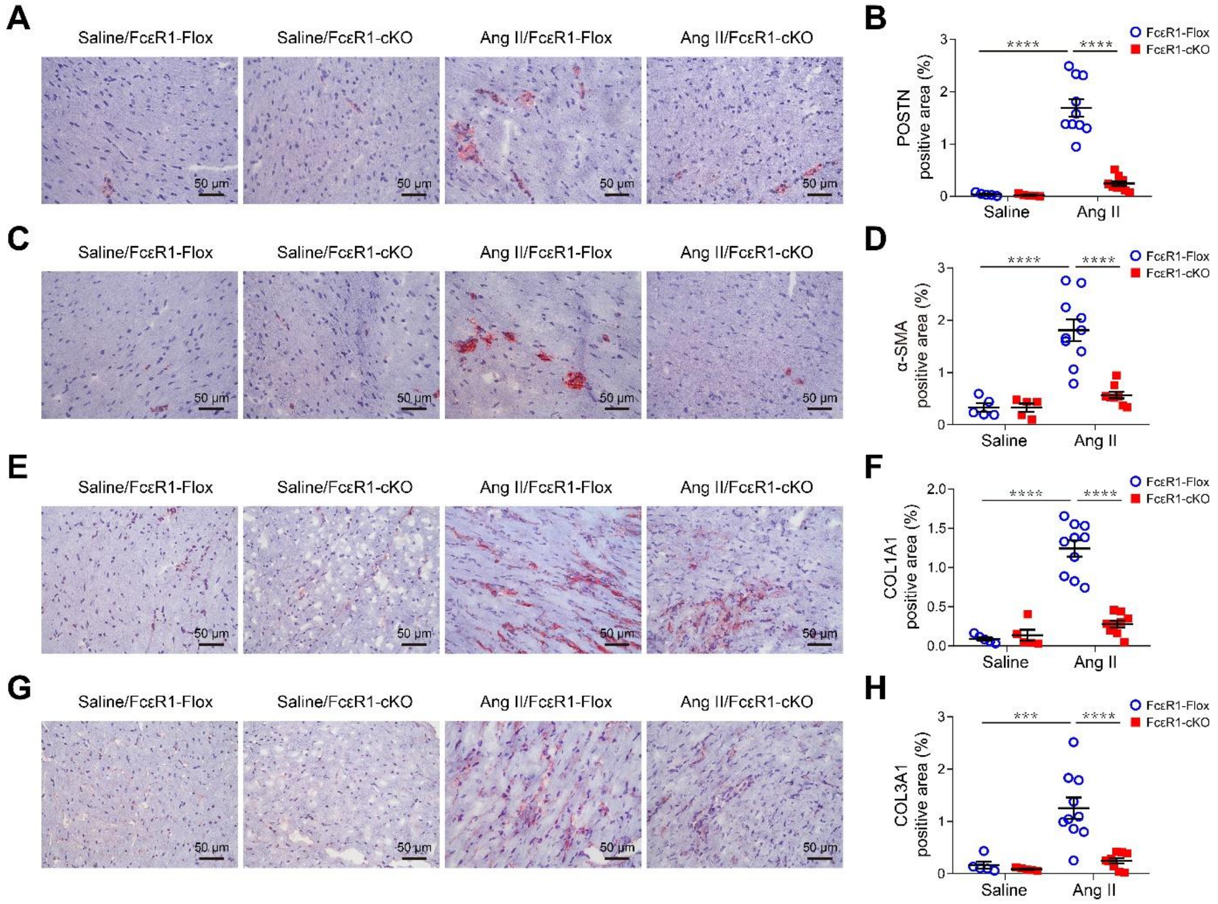


**Figure S5. Immunohistochemical staining of fibrotic markers in heart tissues from CF-specific FcεR1 KO mice**

**A-H.** Representative images of POSTN **(A)**, α-SMA **(C)**, COL1A1 **(E)** and COL3A1 **(G)** staining in heart tissues from Ang II- or Saline-infused FcεR1-cKO or FcεR1-Flox mice. Images were taken at 400X magnification. Scale bars, 50 μm. Quantification of POSTN **(B)**, α-SMA **(D),** COL1A1 **(F)** and COL3A1 **(H)** staining. A total of nine fields from three sections (three fields from each section) per mouse were randomly selected for analysis. Total n = 5 (Saline/FcεR1-Flox), n = 5 (Saline/FcεR1-cKO), n = 10 (Ang II/FcεR1-Flox) or n = 9 (Ang II/FcεR1-cKO) per group. The results are shown as mean ± SEM. ****p* < 0.001, *****p* < 0.0001, n.s. indicates no significance in *Two-way ANOVA* with Bonferroni's post hoc test.

**Figure S6**

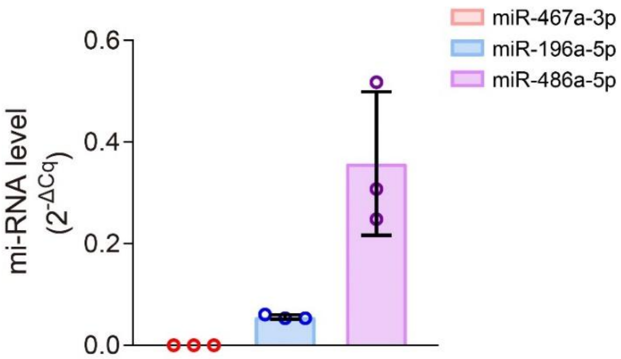


**Figure S6. Basal levels of miRNAs in CFs**

Basal expressions of three candidate miRNAs (miR-467a-3p, miR-196a-5p, and miR-486a-5p) in CFs detected by qPCR. Results are shown as mean ± SD. Data are mean ± SD from 3 independent experiments.

**Figure S7**

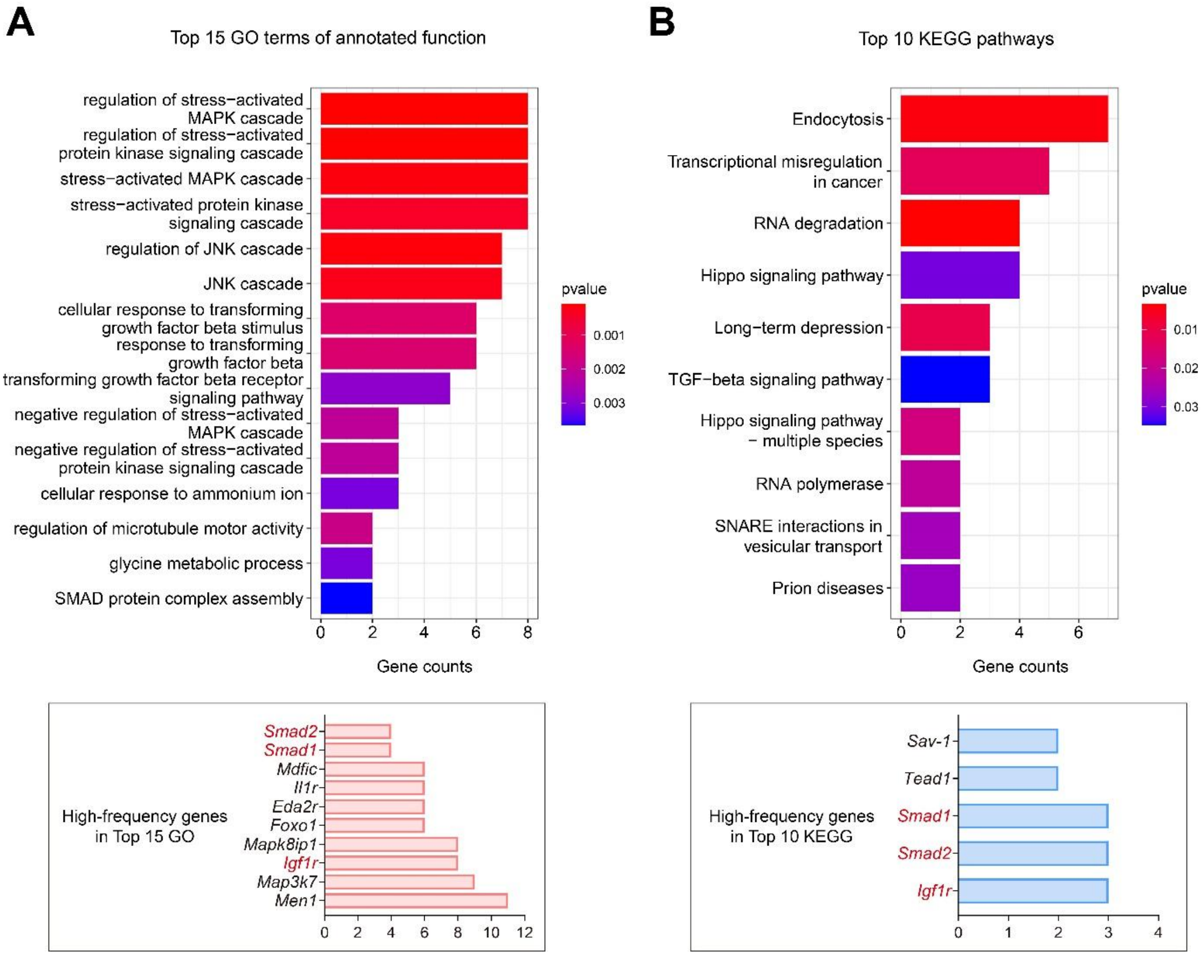


**Figure S7. Gene ontology and KEGG Pathway enrichment analyses for screening potential miR-486a-5p targets**

**A–B.** The predicted candidates from the Targetscan7.1 and miRanda intersection were analyzed by GO and KEGG pathway bioinformatic analyses. **(A)** Upper panel: bar chart of the top fifteen GO terms, listed by -log 10 *p* value. The x-axis shows the gene counts in each GO term and y-axis shows GO terms. Lower panel: The high-frequency genes enriched in top 15 GO. **(B)** upper panel: The enriched KEGG pathways of predicted targets of miR-486a-5p. The x-axis shows the gene counts in each KEGG pathway and y-axis shows KEGG pathways. Lower panel: The high-frequency genes enriched in top 10 KEGG pathways. By overlapping the results from GO and KEGG analyses, three genes (*Smad1*, *Smad2* and *Igf1r*) were identified and marked in red.

**Figure S8**

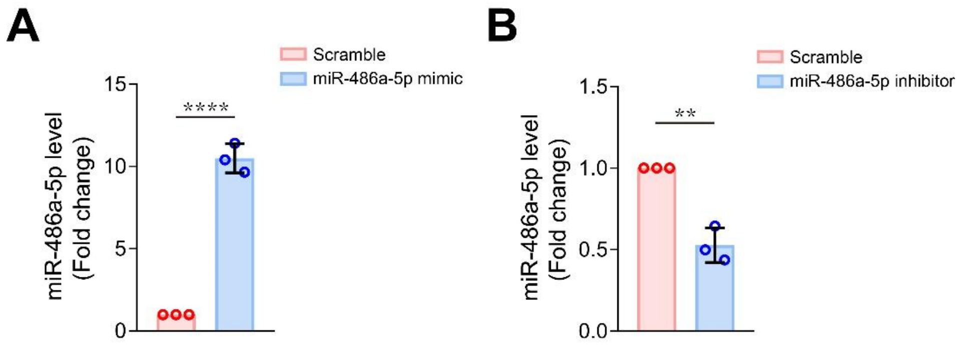


**Figure S8. Efficiency data for miR-486a-5p mimic and miR-486a-5p inhibitor**

**A–B.** Expression of miR-486a-5p in CFs after transfected with miR-486a-5p mimic **(A)** or miR-486a-5p inhibitor **(B)** detected by qPCR (fold change versus Scramble controls). Data are mean ± SD from 3 independent experiments. All statistics were performed using Student's *t*-test. ****$p < 0.0001$, **$p < 0.01$.

Figure S9

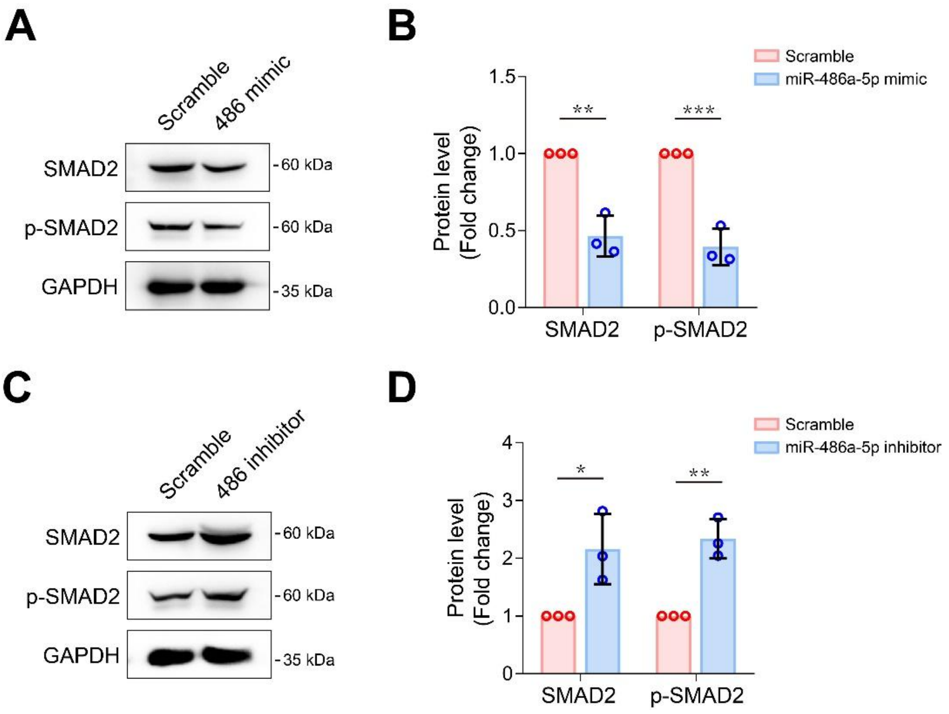


**Figure S9. Expression of SMAD2 and phospho-SMAD2 after miR-486a-5p overexpression or knockdown in CFs**

**A–B.** Representative immunoblot **(A)** and quantification analysis **(B)** of SMAD2 and phospho-SMAD2 expression in CFs after transfected with miR-486a-5p mimic or scrambled control. **C–D.** Representative immunoblot **(C)** and quantification analysis **(D)** of SMAD2 and phospho-SMAD2 in CFs after transfected with miR-486a-5p inhibitor or scrambled control. Data are mean ± SD from 3 independent experiments. All statistics were performed using Student's *t*-test. *$p < 0.05$, **$p < 0.01$, ***$p < 0.001$.

**Figure S10**

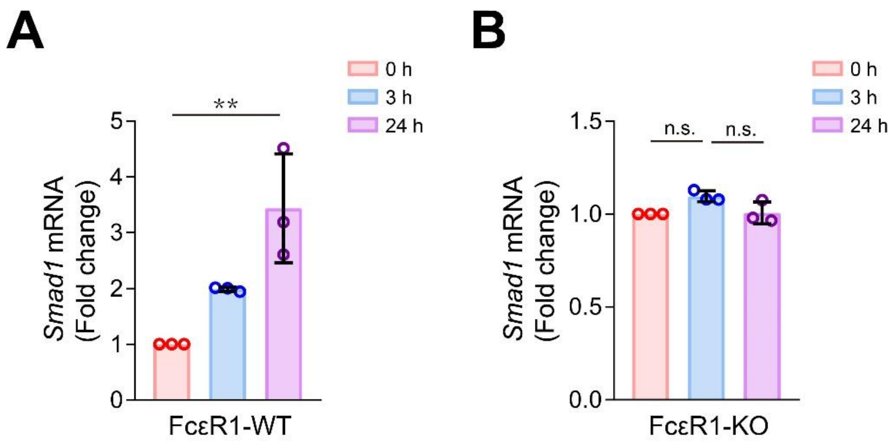


**Figure S10. *Smad1* mRNA expression after IgE stimulation in CFs**

**A–B.** qPCR analysis of *Smad1* mRNA expression in IgE-stimulated FcεR1-WT (A) and FcεR1-KO (B) CFs at indicated times (0, 3, 24 h). Data are mean ± SD from 3 independent experiments. ***p* < 0.01, n.s. indicates no significance in *One-way ANOVA* with Bonferroni's post hoc test.

## Figure S11

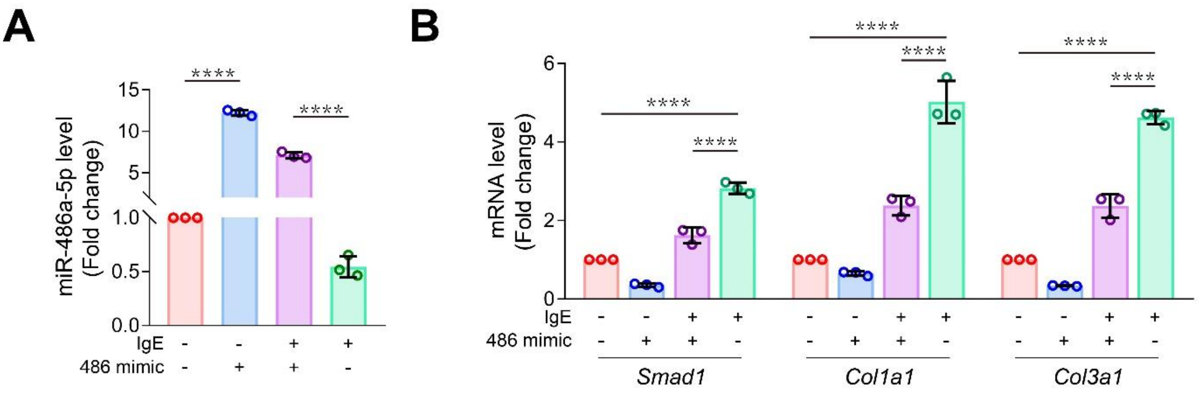


**Figure S11. Rescue assays performed in CFs**

**A–B.** CFs were transfected with miR-486a-5p mimic or scrambled control for 24 hours and then treated with IgE for another 24 hours. qPCR analysis of miR-486a-5p **(A)**, *Smad1*, *Col1a1*, and *Col3a1* **(B)** expression. Data are mean ± SD from 3 independent experiments. All statistics were performed using *Two-way ANOVA* with Bonferroni's post hoc test. ****$p < 0.0001$.

Figure S12

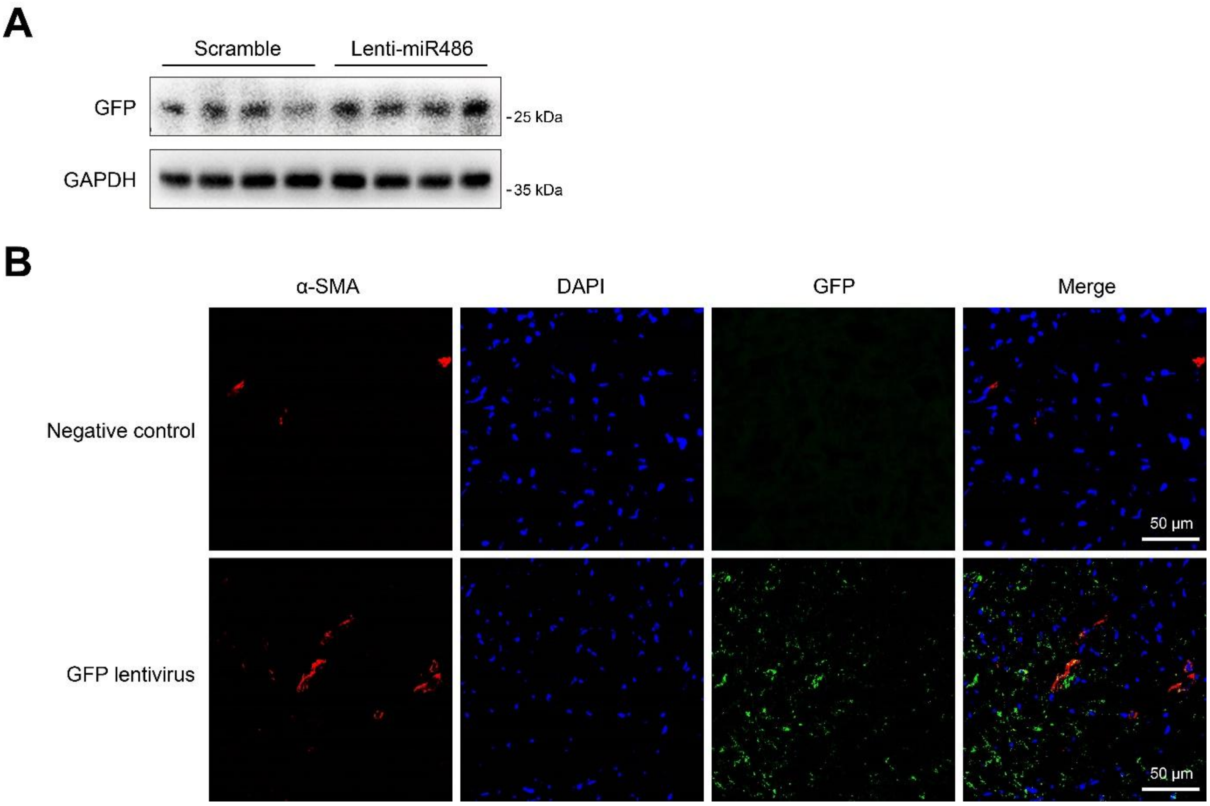


**Figure S12. Overexpression of lenti-miR486 indicated by GFP detection**

**A.** Immunoblot analysis of GFP expression to verify that the lentiviruses were delivered successfully to the heart tissue. **B.** Representative images of immunofluorescence analysis of α-SMA (red), GFP (green) and DAPI (blue) on the heart sections from GFP lentivirus-injected mice and negative controls. Scale bars, 50 μm. DAPI, 4’6-diamidino-2-phenylindole.

**Figure S13**

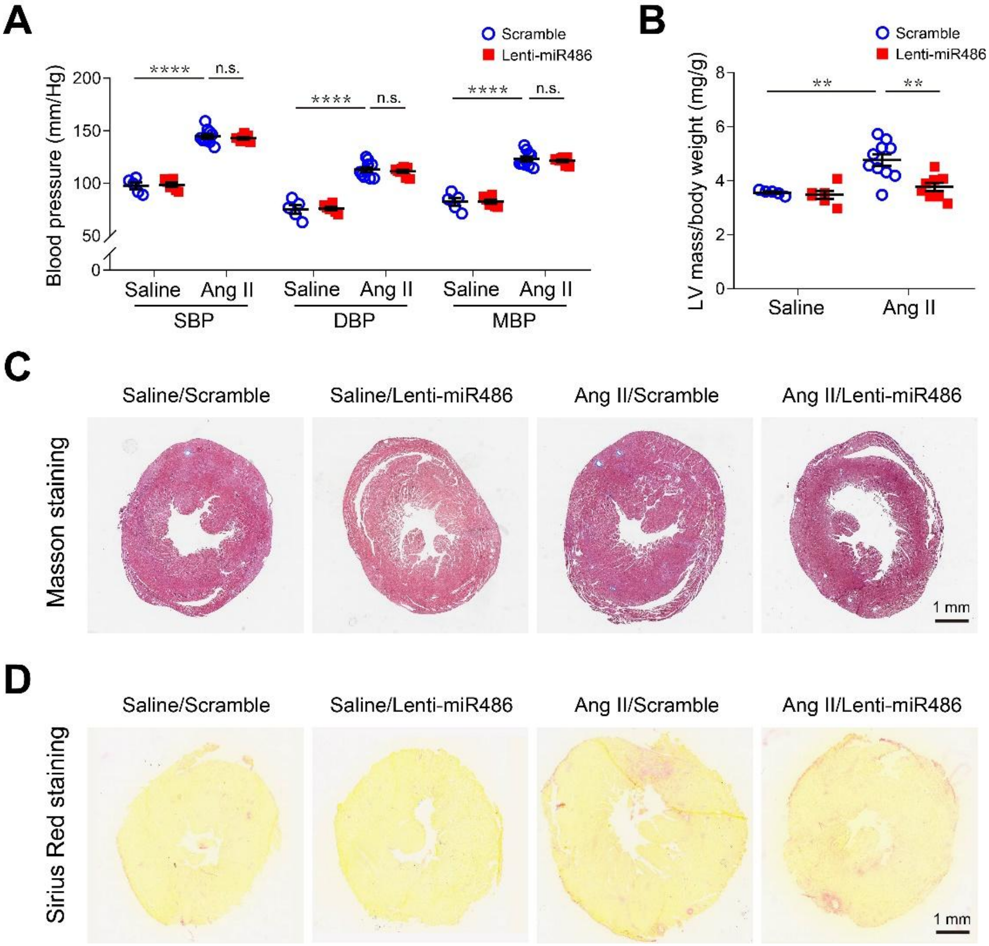


**Figure S13. Effect of miR-486a-5p overexpression on Ang II-infused mice**

**A.** Systolic blood pressure (SBP), diastolic blood pressure (DBP), and mean blood pressure (MBP) were measured by non-invasive tail-cuff monitor in lenti-miR486 or scramble-treated Ang II- or saline-infused mice. **B.** Left ventricular weight versus body weight after lenti-miR486 or scramble-treated Ang II-or saline-infused mice. **C–D.** Representative heart sections examined by Masson **(C)** and Sirius Red staining **(D)**. Scale bars, 1 mm. Total n = 5 (Saline/Scramble), n = 6 (Saline/Lenti-miR486), n = 10 (Ang II/Scramble), or n = 8 (Ang II/Lenti-miR486) per group. The results are shown as mean ± SEM. ***$p < 0.01$, *****$p < 0.0001$, n.s. indicates no significance in *Two-way* ANOVA with Bonferroni's post hoc test.

## Figure S14

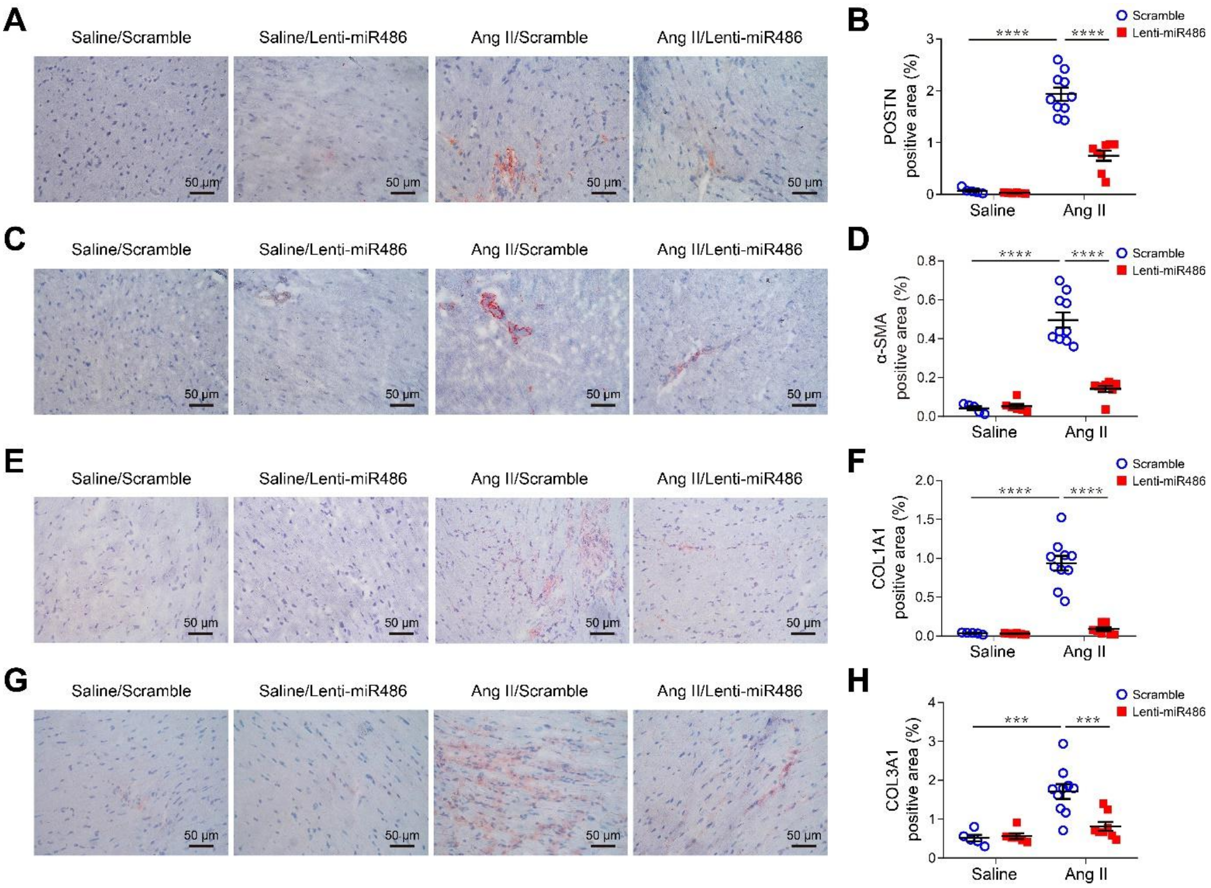


**Figure S14. Immunohistochemical staining of fibrotic markers in heart tissues from miR-486a-5p-overexpressed mice**

**A-H.** Representative images of POSTN **(A)**, α-SMA **(C)**, COL1A1 **(E)**, and COL3A1 **(G)** staining of heart tissues from lenti-miR-486a-5p (lenti-miR486) and scramble treated Ang II- or saline-infused WT mice. Images were taken at 400X magnification. Scale bars, 50 μm. Quantification of POSTN **(B)**, α-SMA **(D)**, COL1A1 **(F)**, and COL3A1 **(H)** staining. A total of nine fields from three sections (three fields from each section) per mouse were randomly selected for analysis. Total n = 5 (Saline/Scramble), n = 6 (Saline/Lenti-miR486), n = 10 (Ang II/Scramble), or n = 8 (Ang II/Lenti-miR486) per group. The results are shown as mean ± SEM. ***$p < 0.001$, ****$p < 0.0001$, n.s. indicates no significance in *Two-way* ANOVA with Bonferroni's post hoc test.

**Figure S15**

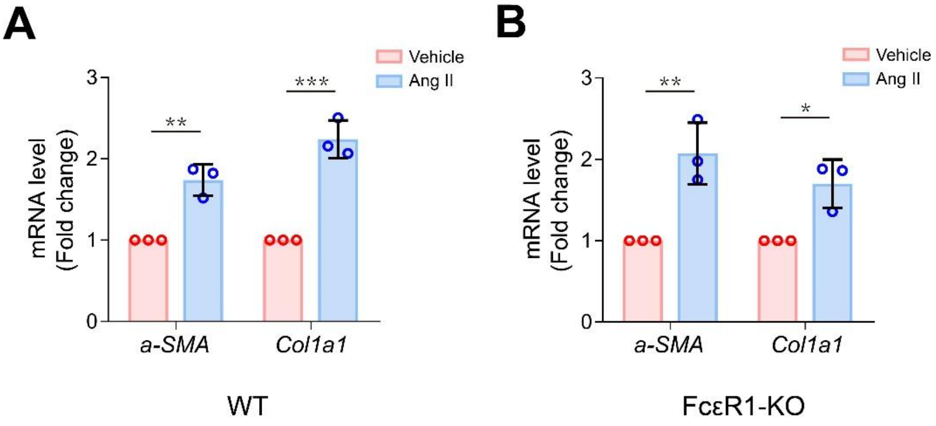


**Figure S15. Effect of Ang II on WT and FcεR1-KO CFs *in vitro***

**A-B.** qPCR analysis of *a-SMA* and *Col1a1* mRNA expression in WT **(A)** and FcεR1-KO **(B)** CFs after IgE after IgE treatment for 24h. Results are shown as mean±SD. **p* < 0.05, ***p* < 0.01, ****p* < 0.001 by Student's *t*-test.

**Figure S16**

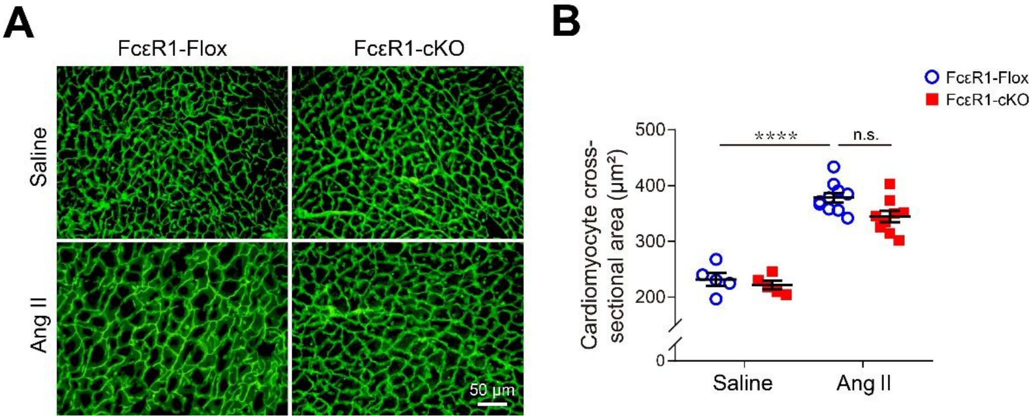


**Figure S16. Effect of CF FcεR1 deletion on Ang II-induced cardiomyocyte hypertrophy**

**A-B.** WGA (green) staining of cardiac sections (6 µm) from Ang II- or saline-infused FcεR1-Flox and FcεR1-cKO mice. 450 cells per mouse were randomly selected from 9 fields in three sections (three random fields from each section) were measured. Scale bars, 50 µm. Total n = 5 (Saline/FcεR1-Flox), n = 5 (Saline/cKO), n = 10 (Ang II/FcεR1-Flox), or n = 9 (Ang II/cKO) per group. Results are shown as mean ± SEM. ****$p < 0.0001$, n.s indicates no significance in *Two-way ANOVA* with Bonferroni's post hoc test.

**Figure S17**

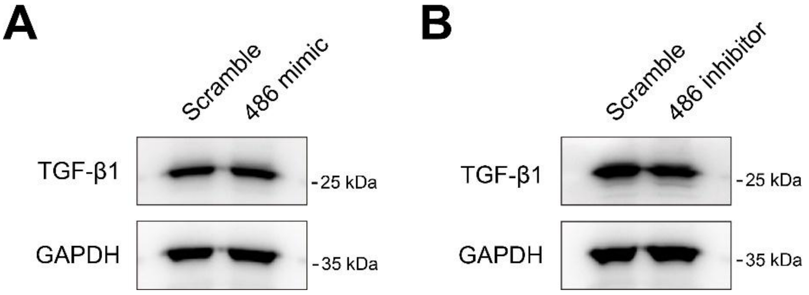


**Figure S17. Expression of TGF-β after miR-486a-5p overexpression or knockdown in CFs**

**A.** Western blot analysis of TGF-β1 protein expression in CFs after transfected with miR-486a-5p mimic or scrambled control. **B.** Western blot analysis of TGF-β protein expression in CFs after transfected with miR-486a-5p inhibitor or scrambled control.

## Supplementary Tables

**Table S2. Echocardiographic analysis of Ang II- or saline-infused FcεR1-Flox and FcεR1-cKO mice**

| | **Saline-treated** | | **Ang II-treated** | |
|---|---|---|---|---|
| | **FcεR1-Flox mice (n = 5)** | **FcεR1-cKO mice (n = 5)** | **FcεR1-Flox mice (n = 10)** | **FcεR1-cKO mice (n = 9)** |
| **LVAW;d (mm)** | 0.76 ± 0.01 | 0.75 ± 0.02 | 1.11 ± 0.02$^{††††}$ | 0.90 ± 0.04**** |
| **LVAW;s (mm)** | 1.17 ± 0.04 | 1.08 ± 0.06 | 1.59 ± 0.03$^{††††}$ | 1.37 ± 0.06** |
| **LVID;d (mm)** | 3.99 ± 0.14 | 4.05 ± 0.09 | 3.65 ± 0.11 | 3.94 ± 0.07 |
| **LVID;s (mm)** | 2.80 ± 0.09 | 2.88 ± 0.07 | 2.64 ± 0.07 | 2.93 ± 0.10 |
| **LVPW;d (mm)** | 0.75 ± 0.01 | 0.82 ± 0.04 | 1.18 ± 0.08$^{††}$ | 0.97 ± 0.04 |
| **LVPW;s (mm)** | 1.04 ± 0.02 | 1.14 ± 0.10 | 1.42 ± 0.09$^{†}$ | 1.22 ± 0.04 |
| **EF (%)** | 57.62 ± 0.55 | 56.34 ± 1.03 | 54.04 ± 2.70 | 50.97 ± 2.73 |
| **FS (%)** | 29.89 ± 0.42 | 29.08 ± 0.68 | 27.57 ± 1.69 | 25.75 ± 1.65 |
| **LV mass AW** | 108.85 ± 4.73 | 117.71 ± 5.11 | 169.02 ± 6.31$^{††††}$ | 142.48 ± 6.82* |
| **LV Mass (correct)** | 87.08 ± 3.78 | 94.17 ± 4.09 | 135.22 ± 5.05$^{††††}$ | 113.98 ± 5.46* |
| **Heart rate (HR, beats/min)** | 498.80 ± 15.14 | 516.80 ± 16.33 | 482.40 ± 10.76 | 504.22 ± 10.83 |

The results are shown as mean ± SEM.

Ang II-treated FcεR1-cKO versus Ang II-treated FcεR1-Flox mice, *$p < 0.05$, **$p < 0.01$, ****$p < 0.0001$.

Ang II-treated FcεR1-Flox mice versus Saline-treated FcεR1-Flox mice, $^{†}p < 0.05$, $^{††}p < 0.01$, $^{††††}p < 0.0001$.

Abbreviations: LVAW;d: left ventricular anterior wall thickness in diastole; LVAW;s: left ventricular anterior wall thickness in systole; LVID;d: left ventricular internal diameter in diastole; LVID;s: left ventricular internal diameter in systole; LVPW;s: left ventricular posterior wall thickness in systole; LVPW;d: left ventricular posterior wall thickness in diastole. EF: ejection fraction; FS: fraction shortening; LV mass AW: left ventricle mass anterior wall. LV Vol;d: left ventricular volume in diastole ; LV vol;s: left ventricular volume in systole.

**Table S6. Echocardiographic analysis of Ang II- or saline-infused WT mice treated with lenti-miR-486a-5p or scramble**

| | Saline-treated | | Ang II-treated | |
|---|---|---|---|---|
| | Scramble (n = 5) | Lenti-miR-486a-5p (n = 6) | Scramble (n = 10) | Lenti-miR-486a-5p (n = 8) |
| **LVAW;d (mm)** | 0.82 ± 0.06 | 0.87 ± 0.05 | 1.07 ± 0.05† | 0.84 ± 0.04** |
| **LVAW;s (mm)** | 1.17 ± 0.07 | 1.25 ± 0.09 | 1.59 ± 0.05†† | 1.19 ± 0.09** |
| **LVID;d (mm)** | 3.72 ± 0.08 | 3.74 ± 0.13 | 3.54 ± 0.10 | 3.79 ± 0.13 |
| **LVID;s (mm)** | 2.50 ± 0.08 | 2.50 ± 0.14 | 2.34 ± 0.11 | 2.71 ± 0.19 |
| **LVPW;d (mm)** | 0.82 ± 0.03 | 0.80 ± 0.04 | 1.01 ± 0.05 | 0.84 ± 0.04 |
| **LVPW;s (mm)** | 1.13 ± 0.03 | 1.24 ± 0.05 | 1.41 ± 0.08 | 1.26 ± 0.06 |
| **EF (%)** | 62.13 ± 1.81 | 62.59 ± 2.62 | 63.39 ± 3.45 | 55.44 ± 4.77 |
| **FS (%)** | 32.89 ± 1.24 | 33.34 ± 1.88 | 34.17 ± 2.44 | 28.89 ± 2.97 |
| **LV mass AW** | 107.95 ± 1.97 | 110.42 ± 4.80 | 140.96 ± 6.73†† | 114.83 ± 5.86* |
| **LV Mass (correct)** | 86.36 ± 1.58 | 88.34 ± 3.84 | 112.77 ± 5.38†† | 91.86 ± 4.69* |
| **Heart rate (HR, beats/min)** | 515.20 ± 7.61 | 509.33 ± 12.71 | 502.80 ± 10.30 | 527.25 ± 6.07 |

The results are shown as mean ± SEM.

Ang II-treated Scramble group versus Ang II-treated Lenti-miR-486a-5p group, *$p < 0.05$, **$p < 0.01$.

Ang II-treated Scramble group versus Saline-treated Scramble group, †$p < 0.05$, ††$p < 0.01$.

Abbreviations: LVAW;d: left ventricular anterior wall thickness in diastole; LVAW;s: left ventricular anterior wall thickness in systole; LVID;d: left ventricular internal diameter in diastole; LVID;s: left ventricular internal diameter in systole; LVPW;s: left ventricular posterior wall thickness in systole; LVPW;d: left ventricular posterior wall thickness in diastole. EF: ejection fraction; FS: fraction shortening; LV mass AW: left ventricle mass anterior wall. LV Vol;d: left ventricular volume in diastole ; LV vol;s: left ventricular volume in systole.
